# Age influences the olfactory profiles of the migratory oriental armyworm *mythimna separate* at the molecular level

**DOI:** 10.1186/s12864-016-3427-2

**Published:** 2017-01-05

**Authors:** Yue-qiu He, Bo Feng, Qian-shuang Guo, Yongjun Du

**Affiliations:** 1 Institute of Health and Environmental Ecology, Wenzhou Medical University, University Town, Wenzhou, 325035 China; 2 Ningbo City College of Vocational Technology, Xuefu Road, Yinzhou High Educational Park, NingBo, 315100 ZheJiang China

**Keywords:** Mythimna separata, Migration, Olfaction, Pre-mating status, Co-receptor, Sex pheromone, Host searching

## Abstract

**Background:**

The oriental armyworm *Mythimna separata* (Walk) is a serious migratory pest; however, studies on its olfactory response and its underlying molecular mechanism are limited. To gain insights to the olfactory mechanism of migration, olfactory genes were identified using antennal transcriptome analysis. The olfactory response and the expression of olfactory genes for 1-day and 5-day-old moths were respectively investigated by EAG and RT-qPCR analyses.

**Results:**

Putative 126 olfactory genes were identified in *M. separata*, which included 43 ORs, 13 GRs, 16 IRs, 37 OBPs, 14 CSPs, and 3 SNMPs. RPKM values of IR75d and 10 ORs were larger than co-receptors IR25a and ORco, and the RPKM value of PR2 was larger than that of other ORs. Expression of GR1 (sweet receptor) was higher than that of other GRs. Several sex pheromones activated evident EAG responses where the responses of 5-day-old male moths to the sex pheromones were significantly greater than those of female and 1-day old male moths. In accordance with the EAG response, 11 pheromone genes, including 6 PRs and 5 PBPs were identified in *M. separate*, and the expression levels of 7 pheromone genes in 5-day-old moths were significantly higher than those of females and 1-day-old moths. PR2 and PBP2 might be used in identifying Z11-16: Ald, which is the main sex pheromone component of *M. separata*. EAG responses to 16 plant volatiles and the expression levels of 43 olfactory genes in 1-day-old moths were significantly greater than that observed in the 5-day-old moths. Heptanal, Z6-nonenal, and benzaldehyde might be very important floral volatiles for host searching and recognized by several olfactory genes with high expression. Some plant volatiles might be important to male moths because the EAG response to 16 plant volatiles and the expression of 43 olfactory genes were significantly larger in males than in females.

**Conclusions:**

The findings of the present study show the effect of adult age on olfactory responses and expression profile of olfactory genes in the migratory pest *M. separate*.

**Electronic supplementary material:**

The online version of this article (doi:10.1186/s12864-016-3427-2) contains supplementary material, which is available to authorized users.

## Background

The oriental armyworm, *Mythimna separata* (Walk), is a serious pest of rice, maize, sorghum, and wheat in China, Japan, Southeast Asia, India, Eastern Australia, New Zealand, and some Pacific Islands [1, 2]. Long-distance movement of *M. separata* between overwintering sites and non-overwintering sites were responsible for six nationwide economic pests in China from 1970 to 1978 [3]. The environmental, physiological, hormonal, and genetic control of individual *M. separata* migratory behavior has been systematically elucidated [3–7]. However, our understanding of olfaction systems in *M. separate* is limited.

Olfaction plays a key role in the interaction of moths with their environment such as foraging, aggregation, mating, and oviposition behaviors. *M. separata* moths utilize olfaction to find nectar, which is used as a supplementary nutrient for egg-ripening [8] and as an energy supplement for flight [9]. Sex pheromones have been used to identify conspecific partners of *M. separata* for mating and its female sex pheromone has been identified [10, 11]. In addition, host plant volatiles but not light conditions significantly affect the level of nocturnal activity in *M. separata* caterpillars [12, 13]. Only a few studies on *M. separata* olfaction have been conducted, which include the antennal sensilla type [14], the EAG response to sex pheromone [15], and the remote-sensing sex pheromone trap for real-time monitoring of *M. separata* [16].

Recently, there has been significant progress in identifying olfactory genes in moths such as *Bombyx mori* [17–19], *Manduca sexta* [20], *Helicoverpa armigera* and *H. assulta* [21], and *Spodoptera frugiperda* [22] and *S. litura* [23]. To date, only three ORs (two PRs and one ORco) and one PBP were identified in *M. separata* antennae [24]. To gain more insights into the molecular mechanism underlying *M. separata* olfaction and into the olfactory mechanism of insect migration, the present study performed antennal *de novo* transcriptome analysis to identify olfactory genes and RT-qPCR to compare the expression profiles between sexes and different pre-mating statuses (1-day- and 5-day-old). The link between OR gene expression and chemosensory responses as measured by electroantennography is also discussed.

## Methods

### Insects

Eggs of *M. separata* were purchased from the Chinese Academy of Agricultural Sciences and were raised in odorless insect incubators with a temperature of 25 ± 1 °C, humidity of 70 ± 7%, and photoperiod (L: D) of 14 h:10 h (from 18:00). Larvae were fed with fresh maize leaves until pupation. Adults were checked at 17:00 daily. Male and female adults were divided into different insect rearing cages (30 cm × 30 cm × 30 cm) and were fed daily with fresh 10% glucose water.

### EAG analysis

Recordings of whole-antenna electrical activity in response to chemicals were performed according to the standard technique described elsewhere [25]. Each chemical (Additional file 1: Table S1) (10 μL) diluted with paraffin oil to concentrations of 10^−2^ and 10^−4^ (V/V) was added onto a filter paper (30 mm × 4 mm) as stimulus, and paraffin oil was used as control. Six antennae of 1-day- and 5-day-old moths were tested after exposure to each chemical for 3.5 h in the dark. As the response of antennae declined during the course of experiment, response to the 1% concentration (V/V) of (*Z*)-3-hexenyl acetate was used as reference and responses to all tested chemicals were standardized.

### Sample collection and total RNA extraction

Antennae of 1-day- and 5-day-old 25 adult *M. separata* (males and females separately) were collected after 3.5 h in the dark. Samples from each group were immediately homogenized in TRNzol-A^+^ (TIANGEN Biotech, Beijing, China) on ice, and total RNA was extracted according to the manual. The concentration and purity of total RNA were determined by using a spectrophotometer NanoDrop2000 (ThermoFisher, Waltham, MA, USA). RNA with an A_260_/A_280_ ratio between 1.75–2.05, an A_260_/A_230_ ratio > 1, and a concentration > 400 ng/μL were used in the following experiments. Extracts were treated with DNase I (Takara, Kusatsu, Shiga, Japan) to remove any DNA. RNA extractions were replicated three times.

### Analysis of de novo transcriptome

The cDNA libraries for transcriptome analysis were prepared using TruSeq SBS Kit v3-HS (Illumina, San Diego, CA, USA) following the manufacturer’s recommendations. Briefly, oligo (dT) magnetic beads were used to isolate poly (A) mRNA from collected total RNA. A fragmentation buffer was added to break the mRNA into short fragments. A random hexamer primer was used to synthesize the first-strand cDNAs using short RNA fragments as templates. The second-strand cDNAs were synthesized using a buffer, dNTPs, RNase H, and DNA polymerase I. After purification, the short cDNAs were linked to sequencing adapters. The libraries were sequenced using a paired-end transcriptome platform Illumina NextSeq500 (Illumina, San Diego, CA, USA) with 2 × 150 bp. Adapters and low-quality bases (base quality < 20) containing reads were discarded from the raw reads to obtain clean reads for analysis. *De novo* transcriptome assembly was conducted with the short reads assembling program, Trinity [26]. BLASTx alignment (E value < 0.00001) between unigenes and protein databases, including NCBI non-redundant protein sequences, Gene Ontology [27], Kyoto Encyclopedia of Genes and Genome [28], eggNOG [29], and SWISS-PROT was successively performed.

### Analysis of olfactory genes

The putative olfactory genes were obtained from gene annotation. Amino acid sequence alignment was performed using CLUSTALX [30]. For the phylogenetic analysis, the amino acid sequences of *Drosophila melanogaster* [31, 32], *H. armigera* [33], *M. sexta* [34], *Ostrinia furnacalis* [35, 36], *Chilo suppressalis* [37], *B. mori* [19, 38], *S. litura* [23], and *Heliothis virescens* [39] were used. Phylogenetic analyses were conducted using the maximum likelihood method of MEGA 6.0, which was based on the Jones-Taylor-Thornton substitution model, partial deletion gaps with 95% site coverage cutoff, and nearest neighbor interchanges heuristic search [40]. Node support of phylogenetic tree was assessed using the bootstrap method with 500 bootstrap replicates.

### Profiling analysis of gene expression based on antennal transcriptome

The gene expression level was calculated using the RPKM method based on the results of antennal transcriptome analysis [41], in which the number of mapped reads per million reads for a gene is divided by the length of that gene. When there were multiple transcript variants for a gene, the longest one was used in calculating its expression level. The identification of differentially expressed genes between male and female antennae was conducted by using DESeq (version 1.18.0) [42]. P-values (p-value < 0.05) and fold-changes (|fold change| > 2) were calculated to determine the differentially expressed genes in the present study.

### RT-qPCR of olfactory gene expression

Single-stranded cDNAs were synthesized from 1 μg of total RNA with ReverTra Ace qPCR RT Kit (Toyobo, Kita-ku, Osaka, Japan) following the manufacturer’s recommendations. qRT-PCR was performed with SsoFast™ EvaGreen® Supermix (Bio-Rad, Hercules, California, USA), following the manufacturer’s protocols, in a CFX-96™ PCR Detection System (Bio-Rad, Hercules, California, USA). The PCR primers used are listed in Additional file 1: Table S2. *Actin* and *AK* were used as reference genes. The difference in gene expression was measured by the 2^-∆∆Ct^ algorithm [43]. For every gene, expressions among all studied tissues were measured with 1-day-old female antennae as control (IR1 with 1-day-old male antennae as control). Then, the logarithm of all data to base 10 was used in generating a heat map. RNA extraction was repeated three times for each sample and two or more RT-qPCR replicates were prepared for each sample.

### Data analysis

Data were analyzed using SPSS 17.0 (SPSS Inc., Chicago, IL, USA). Significance between two samples was determined by using an independent-samples *t*-test. The critical P value for each test was set at 0.05. A heat map was generated by using the software PermutMatrixEN [44].

## Results

### Sequencing and unigene assembly

**Fig. 1:**
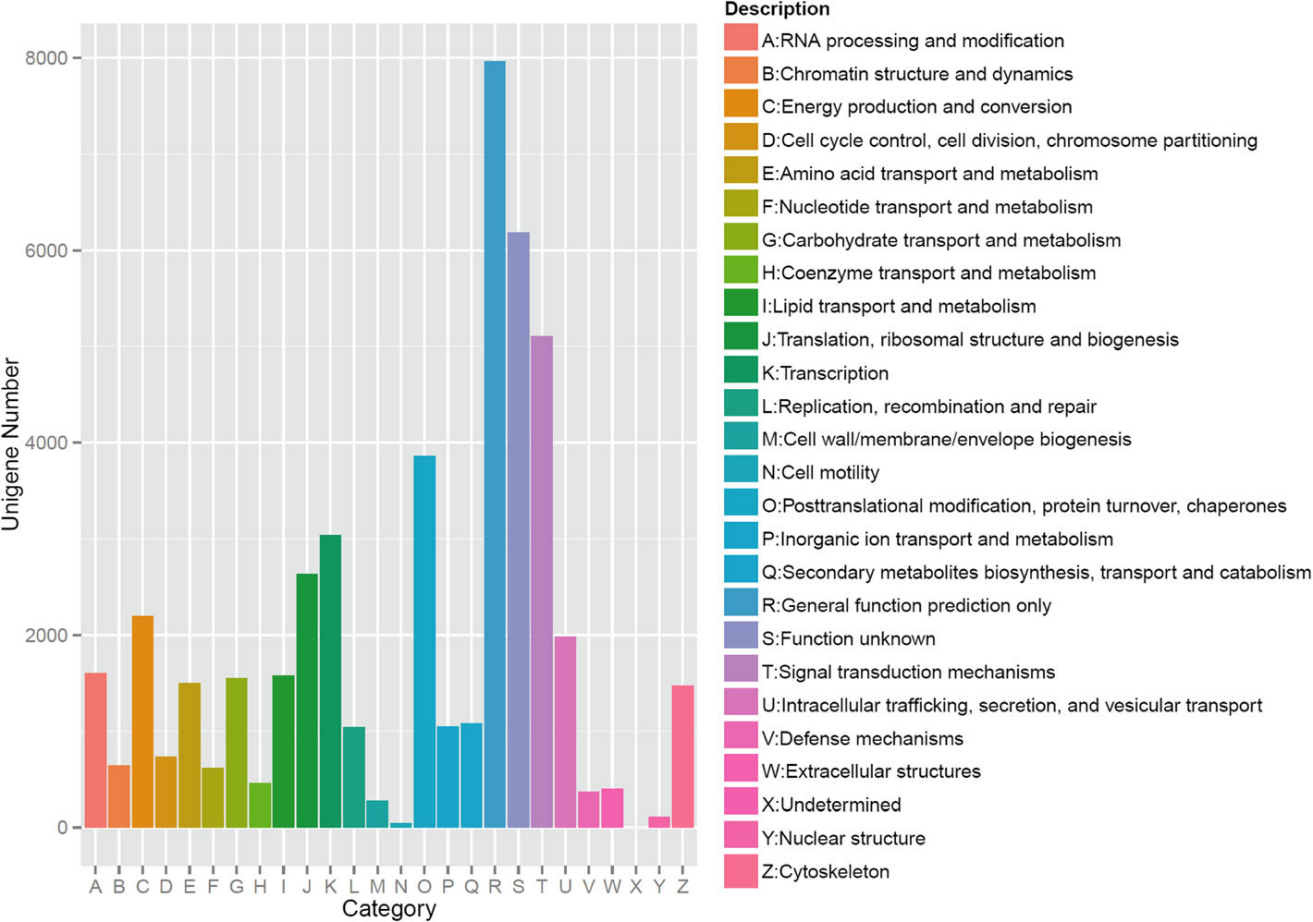
eggNOG annotation of aligned genes from *M. separata* antennal transcriptome. The Y-axis shows the number of the unigene, the X-axis the category of annotation

**Fig. 2:**
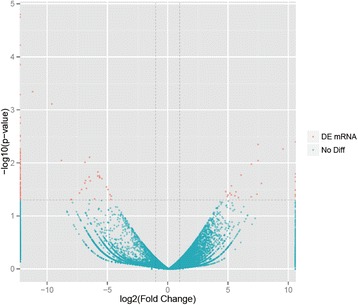
Analysis of different expressed genes (red) in the male and female antennae of *M. separata*. If log2 (fold change) > 1, then this gene is expressed at a higher level in the male antennae; if log2 < −1, then the gene shows a higher expression in the female antennae. The Y-axis shows the p-value of the unigene, the X-axis the fold-change of gene expression (FPKM) in male antennae to female. A total of 28 genes showed a higher expression in the male antennae, and 118 genes were expressed at higher levels in the female antennae. FPKM: fragments per kb per million fragments; DEGs: differentially expressed genes

Using the Illumina NextSeq 500 sequencing system, a total of 75,705,108 (11,355,766,200 bp) and 88,836,476 (12,993,896,231 bp) raw reads were obtained from the female and male samples, respectively. After removing low-quality (<Q20, adaptor and contaminating sequence reads, female and male antennae yielded 69,495,894 (10,045,828,419 bp) and 75,444,012 (11,316,601,660 bp) clean reads, respectively. Pooled female and male data were assembled into 160,299 contigs (N50 = 676 bp), with an average length of 537 nt, and 41,056 unigenes (N50 = 1301) with a mean length of 763 bp (Additional file 2: Figure S1). For the evolutionary genealogy of genes, eggNOG annotation was used to assess the transcriptome (Fig. 1), and 5105 (10.75%) unigenes were assigned to signal transduction mechanisms. Among these unigenes, male antennae showed 28 genes with a higher expression, whereas 118 genes were upregulated in the female antennae (Fig. 2).

### Analysis of olfactory and gustatory genes

Approximately 43 putative OR genes, 13 putative GR genes, 16 putative IR genes, 37 putative OBP genes, 14 CSP genes, and 3 SNMP genes were identified in *M. separata* (Tables 1, 2, 3, 4, 5, and 6).

**Fig. 3:**
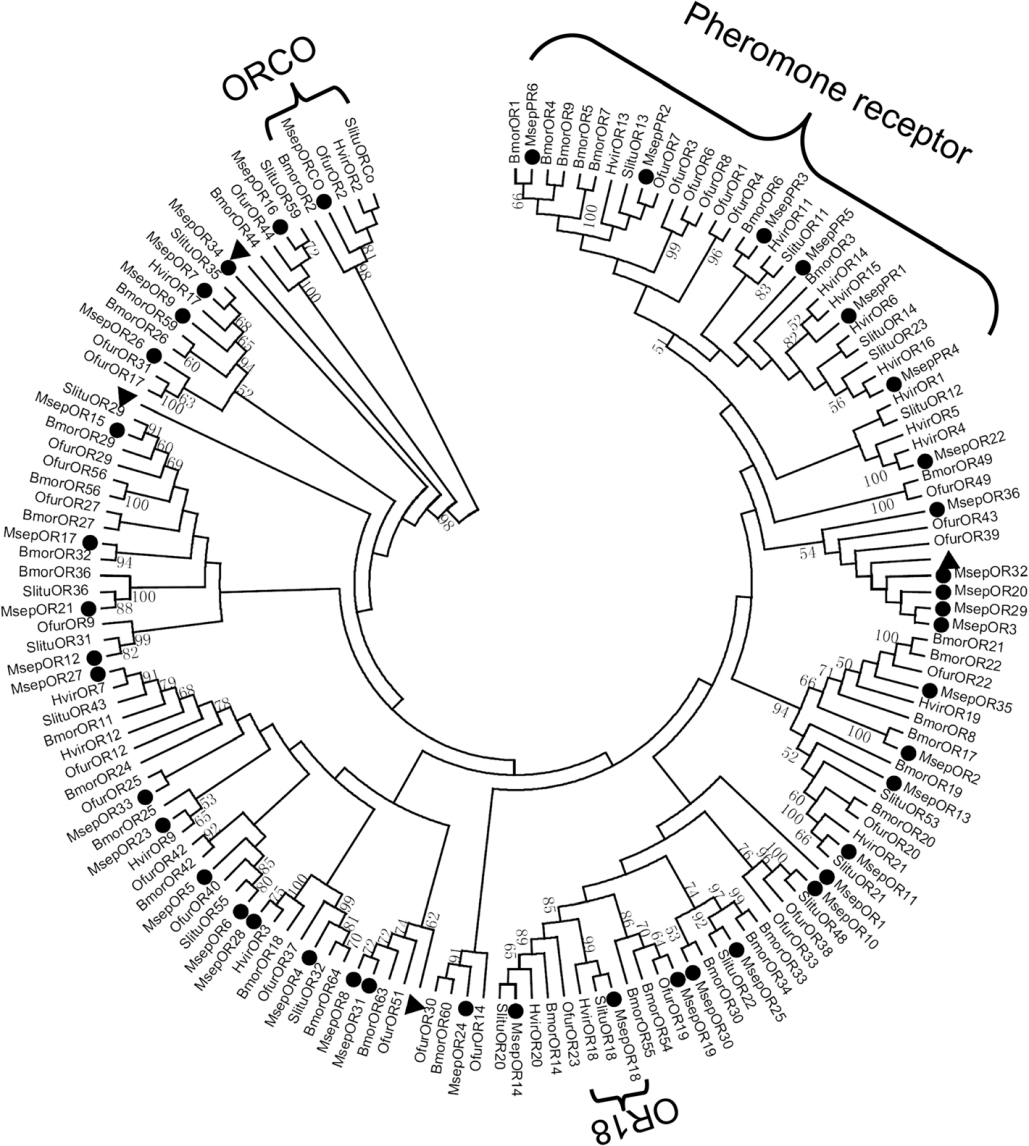
Aligned putative OR gene sequences of *M. separata* (black circle). Bootstrap values < 50% were ignored. Msep, *M. separata*, Bmor, *B. mori*, Hvir, *Heliothis virescens*, Ofur, *O. furnacalis*, Slitu, *S. litura*

The OR genes included one ORco, one OR18 ortholog, 6 PR genes, and 35 general OR genes (Table 1). Eleven ORs were likely full-length genes that encoded proteins of more than 390 amino acids, including PR2, PR3, ORco, OR1, OR3, OR4, OR6-9, and OR12. Four general OR genes (OR1, 5, 33, and 34) were not effectively clustered with other moths ORs (bootstrap values < 50) in the phylogenetic analysis (Fig. 3).

**Table 1:** BLASTP results of candidate olfactory receptors of *M. separata*

Gene name	Protein length (amino acids)	Full ORF	Reference gene name	Reference gene ID	E_value	Similarity (%)
*OR1*	402	Yes	Olfactory receptor 29 [*Operophtera brumata*]	KOB71190	0	73.9
*OR2*	350	No	Olfactory receptor 17 [*Bombyx mori*]	NP_001157210	1.3E-108	45.7
*OR3*	419	Yes	Odorant receptor 38 [*Athetis dissimilis*]	ALM26228	0	85.4
*OR4*	415	Yes	Odorant receptor 65, partial [*Athetis dissimilis*]	ALM26248	0	85.1
*OR5*	373	No	Odorant receptor 4-like [*Bombyx mori*]	XP_012547825	9.9E-180	66.0
*OR6*	452	Yes	Olfactory receptor 12 [*Spodoptera litura*]	AGG08878	0	83.6
*OR7*	390	Yes	Olfactory receptor 17 [*Helicoverpa assulta*]	AGK90020	0	79.0
*OR8*	392	Yes	Odorant receptor 63, partial [*Athetis dissimilis*]	ALM26246	0	82.9
*OR9*	391	Yes	Odorant receptor [*Helicoverpa armigera*]	AIG51873	0	83.4
*OR10*	382	No	Odorant receptor, partial [*Helicoverpa armigera*]	AIG51891	0	85.9
*OR11*	349	No	Olfactory receptor 21, partial [*Helicoverpa assulta*]	AJD81557	0	75.1
*OR12*	433	Yes	Odorant receptor [*Helicoverpa armigera*]	AIG51892	0	81.1
*OR13*	312	No	Odorant receptor [*Helicoverpa armigera*]	AIG51871	8.4E-152	65.7
*OR14*	329	No	Odorant receptor 20 [*Athetis dissimilis*]	ALM26209	0	86.3
*OR15*	242	No	Odorant receptor [*Helicoverpa armigera*]	AIG51879	3.1E-156	87.6
*OR16*	265	No	Odorant receptor 44 [*Athetis dissimilis*]	ALM26234	0	93.2
*OR17*	239	No	Odorant receptor, partial [*Helicoverpa armigera*]	AIG51876	1.3E-136	77.8
*OR18*	132	No	Olfactory receptor 18 [*Mamestra brassicae*]	ACL81188	2.94E-84	93.2
*OR19*	248	No	Odorant receptor [*Dendrolimus houi*]	AII01057	9.47E-54	34.7
*OR20*	212	No	Odorant receptor 36, partial [*Athetis dissimilis*]	ALM26226	3E-113	74.5
*OR21*	210	No	Olfactory receptor 35 [*Manduca sexta*]	CUQ99415	2.3E-101	72.9
*OR22*	196	No	Olfactory receptor 4, partial [*Helicoverpa armigera*]	ACF32962	7.6E-118	86.2
*OR23*	199	No	Chemosensory receptor 9 [*Heliothis virescens*]	CAD31950	1.64E-74	58.8
*OR24*	139	No	Odorant receptor [*Helicoverpa armigera*]	AIG51888	2.39E-77	92.8
*OR25*	165	No	Odorant receptor, partial [*Helicoverpa armigera*]	AIG51880	1.89E-73	63.6
*OR26*	126	No	Olfactory receptor 26, partial [*Helicoverpa assulta*]	AJD81561	6.22E-77	88.9
*OR27*	125	No	Odorant receptor 7 [*Athetis dissimilis*]	ALM26195	6.59E-70	89.6
*OR28*	130	No	Odorant receptor, partial [*Helicoverpa armigera*]	AIG51852	2.21E-77	90.8
*OR29*	135	No	Chemosensory receptor 10 [*Heliothis virescens*]	CAG38111	2.8E-81	90.4
*OR30*	118	No	Odorant receptor 30 [*Athetis dissimilis*]	ALM26219	6.52E-74	92.4
*OR31*	114	No	Olfactory receptor 43, partial [*Helicoverpa assulta*]	AJD81577	3.44E-58	78.9
*OR32*	261	No	Odorant receptor 16 [*Athetis dissimilis*]	ALM26205	1.6E-164	84.7
*OR33*	124	No	Odorant receptor 40 [*Athetis dissimilis*]	ALM26230	2.08E-56	76.6
*OR34*	100	No	Odorant receptor 43a-like [*Bombyx mori*]	XP_012548773	1.66E-33	60.0
*OR36*	97	No	Olfactory receptor 33, partial [*Helicoverpa assulta*]	AJD81568	8.36E-55	85.6
*ORco*	473	Yes	Olfactory receptor-2 [*Mythimna separata*]	BAG71415	0	100.0
*PR1*	120	No	Olfactory receptor-1 [*Mythimna separata*]	BAG71414	2.62E-72	92.5
*PR2*	424	Yes	Olfactory receptor [*Mythimna separata*]	BAG71423	0	99.3
*PR3*	435	Yes	Olfactory receptor 3 [*Agrotis segetum*]	AGS41442	0	88.0
*PR4*	243	No	Odorant receptor [*Sesamia inferens*]	AGY14579	3.5E-146	84.4
*PR5*	99	No	Odorant receptor 1 [*Athetis dissimilis*]	ALM26192	2.9E-46	78.8
*PR6*	103	No	Odorant receptor 36, partial [*Athetis dissimilis*]	ALM26226	3.01E-25	46.6

**Fig. 4:**
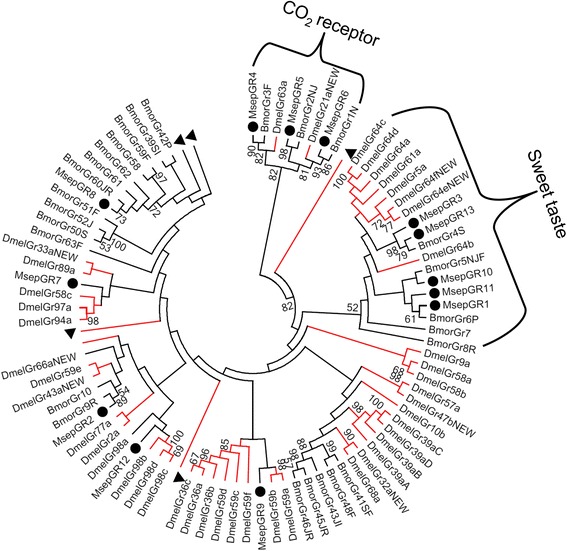
Aligned putative GR gene sequences of *M. separata* (black circle), *D. melanogaster* (red lines) and other moth species (black lines). Bootstrap values < 50% were ignored. Msep, *M. separata*, Dmel, *D. melanogaster*, Bmor, *B. mori*

GR genes included 3 CO_2_ receptors (GR4-6), 5 sweet receptors (GR1, 3, 10, 11, and 13), and 5 bitter taste receptor genes (Table 2). The sequence sizes of GR1 were longer than 440 amino acids, indicating that it is a nearly full-length gene. Two bitter taste receptors (GR2 and GR8) were clustered with the GR genes of *B. mori*, with bootstrap values > 50 in the phylogenetic analysis (Fig. 4).

**Table 2:** BLASTP results of candidate gustatory receptors of *M. separata*

Gene name	Protein length (amino acids)	Reference gene name	Reference gene ID	E_value	Similarity (%)
*GR1*	449	Chemosensory receptor 1 [*Heliothis virescens*]	CAD31850	0	84.9
*GR3*	244	Gustatory receptor 7, partial [*Athetis dissimilis*]	ALM26256	7.1E-142	82.8
*GR2*	282	Gustatory receptor [*Helicoverpa armigera*]	AGA04648	0	94.3
*GR4*	203	Gustatory receptor 3 [*Helicoverpa assulta*]	AJD81596	7.1E-124	94.1
*GR5*	193	Gustatory receptor 2, partial [*Helicoverpa assulta*]	AJD81595	1.3E-133	97.9
*GR6*	134	Gustatory receptor 1, partial [*Helicoverpa assulta*]	AJD81594	3.87E-86	92.5
*GR7*	131	Gustatory receptor [*Helicoverpa armigera*]	AIG51908	4.26E-78	88.5
*GR8*	112	Gustatory receptor 11, partial [*Helicoverpa assulta*]	AJD81604	1.11E-12	31.3
*GR9*	105	Gustatory receptor 11, partial [*Helicoverpa assulta*]	AJD81604	2.79E-11	29.5
*GR10*	110	Gustatory receptor for sugar taste 64f-like [*Bombyx mori*]	XP_012552784	1.09E-14	32.7
*GR11*	85	Gustatory receptor for sugar taste 64f-like [*Amyelois transitella*]	XP_013189983	3.02E-24	55.3
*GR12*	88	Gustatory receptor 8, partial [*Athetis dissimilis*]	ALM26257	1.59E-28	63.6
*GR13*	79	Gustatory receptor 8, partial [*Athetis dissimilis*]	ALM26257	7.36E-39	84.8

**Fig. 5:**
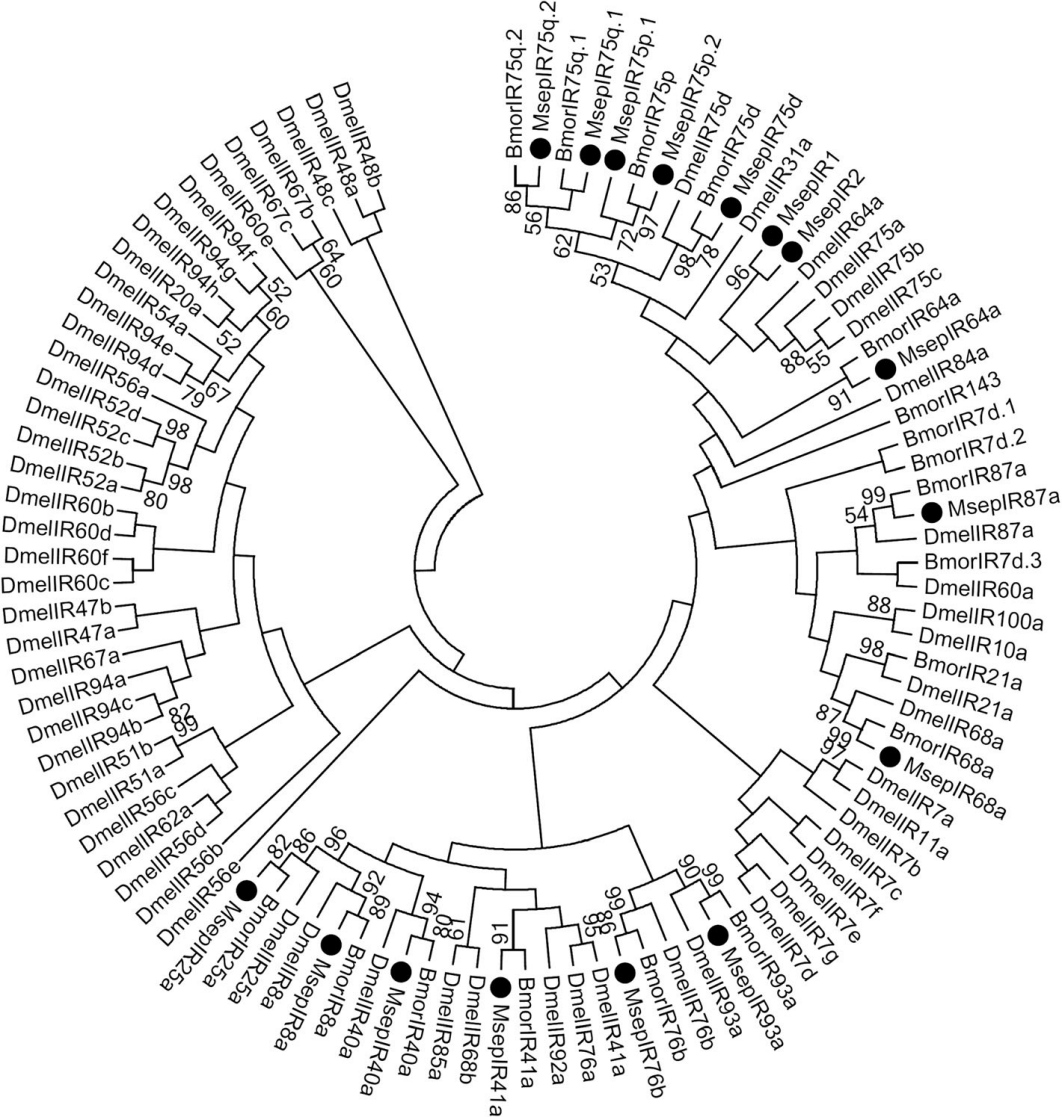
Aligned putative IR gene sequences of *M. separata* (black circle). Bootstrap values < 50% were ignored. Msep, *M. separata*, Dmel, *D. melanogaster*, Bmor, *B. mori*

Fourteen conserved IR genes were identified in *M. separata*, including IR25a, IR40a, IR41a, IR64a, IR68a, IR76b, IR87a, IR8a, IR93a, IR75d, two IR75p genes (IR75p1 and IR75p2), and two IR75q genes (IR75q.1 and IR75q.2). Five IRs were nearly full-length genes that encoded proteins of more than 560 amino acids, including IR75p.2, IR75d, IR75p.1, IR8a, and IR25a. In addition, two diversified IR genes (IR1 and IR2) were identified in *M. separata* (Table 3), and these were not clustered with the IR genes used with bootstrap values > 50 (Fig. 5).

**Table 3:** BLASTP results of candidate ionotropic receptors of *M. separata*

Gene name	Protein length (amino acids)	Reference gene name	Reference gene ID	E_value	Similarity (%)
*IR8a*	842	Ionotropic receptor 8a.1 [*Athetis dissimilis*]	ALM24945	0	91.6
*IR93a*	246	Ionotropic receptor [*Ostrinia furnacalis*]	BAR64811	1.2E-141	78.9
*IR75q.2*	96	Ionotropic receptor 75q.2 [*Athetis dissimilis*]	ALM24940	1.34E-56	94.8
*IR75q.1*	40	Ionotropic receptor 75q.1, partial [*Helicoverpa assulta*]	AJD81638	2.96E-08	65.0
*IR75d*	600	Ionotropic receptor 75d [*Athetis dissimilis*]	ALM24944	0	77.8
*IR87a*	127	Chemosensory ionotropic receptor IR87a [*Spodoptera littoralis*]	ADR64689	2.1E-80	94.5
*IR76b*	500	Ionotropic receptor [*Sesamia inferens*]	AGY49253	0	87.4
*IR75p.2*	568	Ionotropic receptor [*Ostrinia furnacalis*]	BAR64805	0	63.7
*IR1*	196	Chemosensory ionotropic receptor IR1 [*Spodoptera littoralis*]	ADR64688	2.7E-98	75.5
*IR68a*	249	Chemosensory ionotropic receptor IR68a [*Spodoptera littoralis*]	ADR64682	2.8E-143	82.3
*IR40a*	228	Ionotropic receptor 3 [*Athetis dissimilis*]	ALM24948	2.8E-143	93.4
*IR41a*	251	Chemosensory ionotropic receptor IR41a [*Spodoptera littoralis*]	ADR64681	1E-152	86.1
*IR25a*	876	Ionotropic receptor 25a, partial [*Helicoverpa assulta*]	AJD81628	0	97.4
*IR64a*	450	Ionotropic receptor [*Ostrinia furnacalis*]	BAR64801	2E-172	52.7
*IR75p.1*	652	Probable glutamate receptor [*Bombyx mori*]	XP_012551951	0	66.6
*IR2*	366	Ionotropic receptor, partial [*Ostrinia furnacalis*]	BAR64812	2.3E-149	63.9

**Fig. 6:**
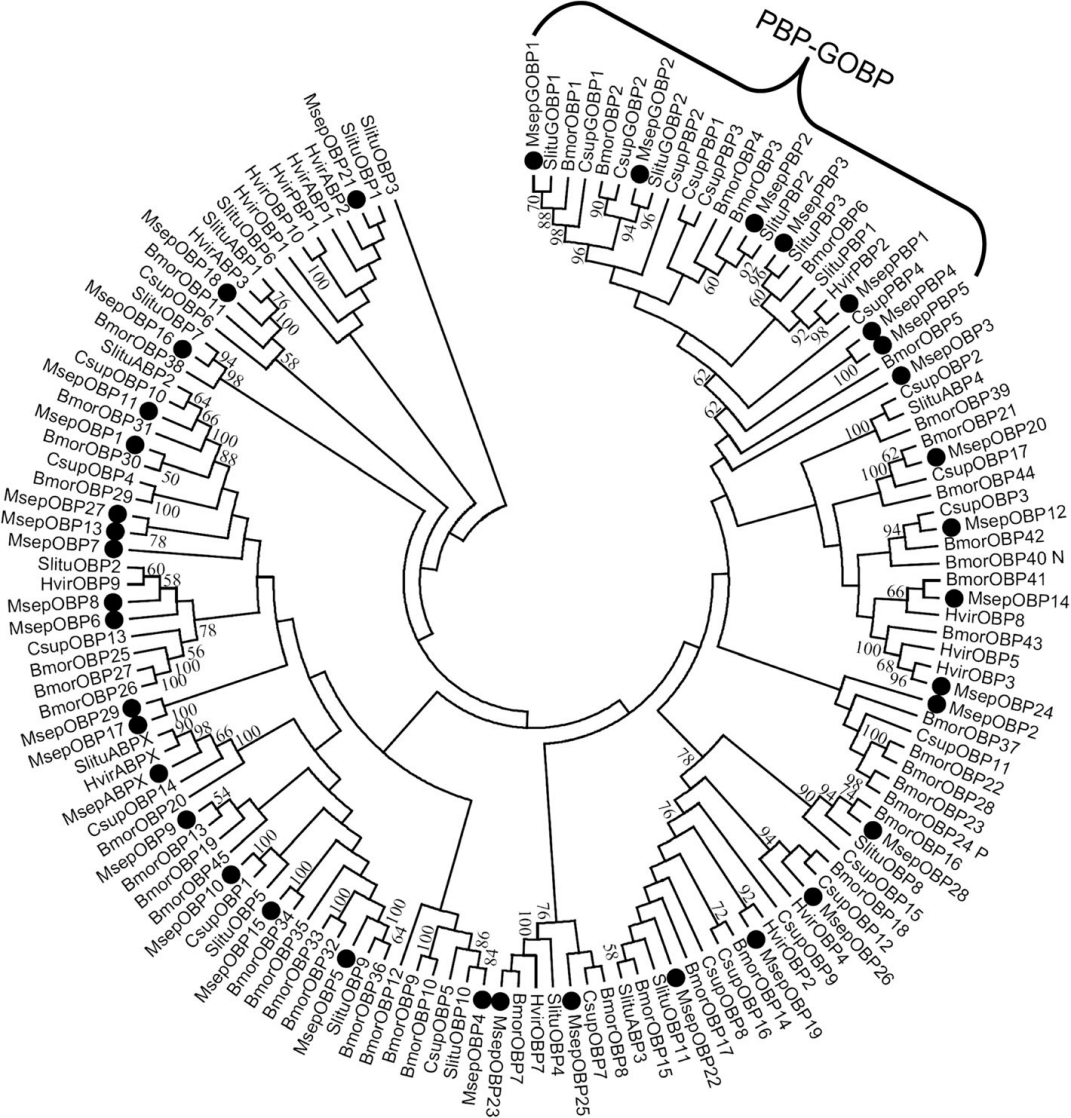
Aligned putative OBP gene sequences of *M. separata* (black circle). Bootstrap values lower than 50% were ignored. Msep, *M. separata*, Bmor, *B. mori*, Slitu, *S. litura*, Hvir, *Heliothis virescens*, Csup, *C. suppressalis*

OBPs included 2 GOBPs (GOBP1-2), ABPX, 5 PBPs (PBP1-5), and 29 OBPs. Except for PBP3, OBP9, OBP4, PBP5, OBP13, and OBP29, other 31 OBPs were likely or nearly full-length genes that encoded proteins of more than 140 amino acids. Around 29 OBPs was subdivided into 16 classic OBP genes, 10 minus-C OBP genes (OBP1, 6–9, 11, 13, 17, 27, and 29), which lack two cysteine residues (C2 and C5), and 3 plus-C OBP genes (OBP12, 14, and 24), which have more than 6 cysteine residues (Table 4, Additional file 2: Figure S2). OBP2, 3, 7, 13, 17, 27, and 29 were not clustered with the moth OBP gene with bootstrap values > 50 in the phylogenetic analysis (Fig. 6).

**Table 4:** BLASTP results of candidate odorant binding proteins of *M. separata*

Gene name	Full ORF	Group	Protein length (amino acids)	Reference gene name	Reference gene ID	E_value	Similarity (%)
*ABPX*	No	C	140	ABPX, partial [*Sesamia inferens*]	AGS36754	5.15E-67	75.0
*GOBP1*	Yes	C	193	General odorant binding protein 1, partial [*Agrotis segetum*]	ABI24159	6.9E-99	72.0
*GOBP2*	Yes	C	162	General odorant binding protein 2 precursor [*Mamestra brassicae*]	AAC05703	5.1E-106	92.0
*OBP1*	Yes	M	145	Odorant-binding protein 5 [*Chilo suppressalis*]	AGK24581	2.8E-18	31.0
*OBP10*	Yes	C	175	Odorant binding protein 1 [*Spodoptera litura*]	AKI87962	9.8E-105	86.3
*OBP11*	Yes	M	133	Odorant binding protein 9 [*Spodoptera exigua*]	AGH70105	2.43E-81	90.2
*OBP12*	Yes	P	197	Odorant-binding protein 19 [*Helicoverpa assulta*]	AGC92793	9.04E-77	60.9
*OBP13*	No	M	105	Odorant binding protein [*Lissorhoptrus oryzophilus*]	AHE13799	2.35E-21	43.8
*OBP14*	Yes	P	183	Odorant binding protein 1, partial [*Agrotis ipsilon*]	AGR39564	1.25E-87	70.5
*OBP15*	Yes	C	182	Odorant-binding protein 2 [*Danaus plexippus*]	EHJ74351	2.5E-111	85.2
*OBP16*	Yes	C	168	Odorant binding protein 4 [*Spodoptera litura*]	AKI87965	4.35E-82	73.2
*OBP17*	Yes	M	133	Odorant-binding protein 2 [*Monochamus alternatus*]	AHA39267	6.5E-90	91.0
*OBP18*	Yes	C	148	Antennal binding protein [*Heliothis virescens*]	CAC33574	4.55E-64	67.6
*OBP19*	Yes	C	142	OBP2 [*Helicoverpa armigera*]	AEB54586	2.76E-86	87.3
*OBP2*	No	C	141	OBP13 [*Sesamia inferens*]	AGS36753	3.28E-08	24.1
*OBP20*	Yes	C	141	OBP16, partial [*Sesamia inferens*]	AGS36756	1.8E-79	83.0
*OBP21*	Yes	C	146	pheromone binding protein 4 [*Mamestra brassicae*]	AAL66739	1.43E-82	83.6
*OBP22*	Yes	C	146	Odorant binding protein 17 [*Spodoptera litura*]	ALD65891	5.28E-82	82.2
*OBP23*	Yes	C	145	Odorant binding protein [*Spodoptera exigua*]	ADY17886	7.5E-80	79.3
*OBP24*	Yes	P	146	Odorant binding protein 6, partial [*Agrotis ipsilon*]	AGR39569	1.71E-76	74.7
*OBP25*	Yes	C	147	Odorant binding protein OBP1 [*Spodoptera litura*]	ALJ30188	7.25E-66	66.0
*OBP26*	Yes	C	139	OBP8 [*Helicoverpa armigera*]	AEB54589	5.74E-85	87.8
*OBP27*	Yes	M	135	Odorant-binding protein 21 [*Dastarcus helophoroides*]	AIX97067	5.63E-46	55.6
*OBP28*	Yes	C	140	SexiOBP10 [*Spodoptera exigua*]	AGP03456	7.22E-70	73.6
*OBP29*	No	M	115	Odorant binding protein 6 [*Monochamus alternatus*]	AJO67868	5.3E-78	90.0
*OBP3*	Yes	C	135	OBP13 [*Sesamia inferens*]	AGS36753	1.04E-21	30.4
*OBP4*	No	C	77	OBP7, partial [*Sesamia inferens*]	AGS36749	3.19E-40	83.1
*OBP5*	Yes	C	192	Odorant-binding protein 2 [*Danaus plexippus*]	EHJ67147	5.6E-99	70.3
*OBP6*	Yes	M	142	Antennal binding protein 7 [*Manduca sexta*]	AAL60425	3.32E-16	38.0
*OBP7*	No	M	178	Antennal binding protein 7 [*Antheraea yamamai*]	ADO95155	1.55E-09	21.9
*OBP8*	Yes	M	138	Odorant binding protein 5 [*Agrotis ipsilon*]	AGR39568	2.87E-64	71.7
*OBP9*	No	M	66	SexiOBP13 [*Spodoptera exigua*]	AGP03459	1.32E-06	31.8
*PBP1*	No	C	142	Pheromone binding protein 1 precursor [*Mamestra brassicae*]	AAC05702	1.44E-83	84.5
*PBP2*	Yes	C	170	Pheromone binding protein [*Mythimna separata*]	BAG71416	3.3E-119	98.2
*PBP3*	No	C	64	Pheromone-binding protein 3 [*Agrotis ipsilon*]	AFM36758	2.64E-31	85.9
*PBP4*	Yes	C	195	PBP2 [*Helicoverpa armigera*]	AEB54583	4.43E-21	24.1
*PBP5*	No	C	100	Pheromone binding protein 1 [*Danaus plexippus*]	EHJ71307	4.07E-05	24.0

**Fig. 7:**
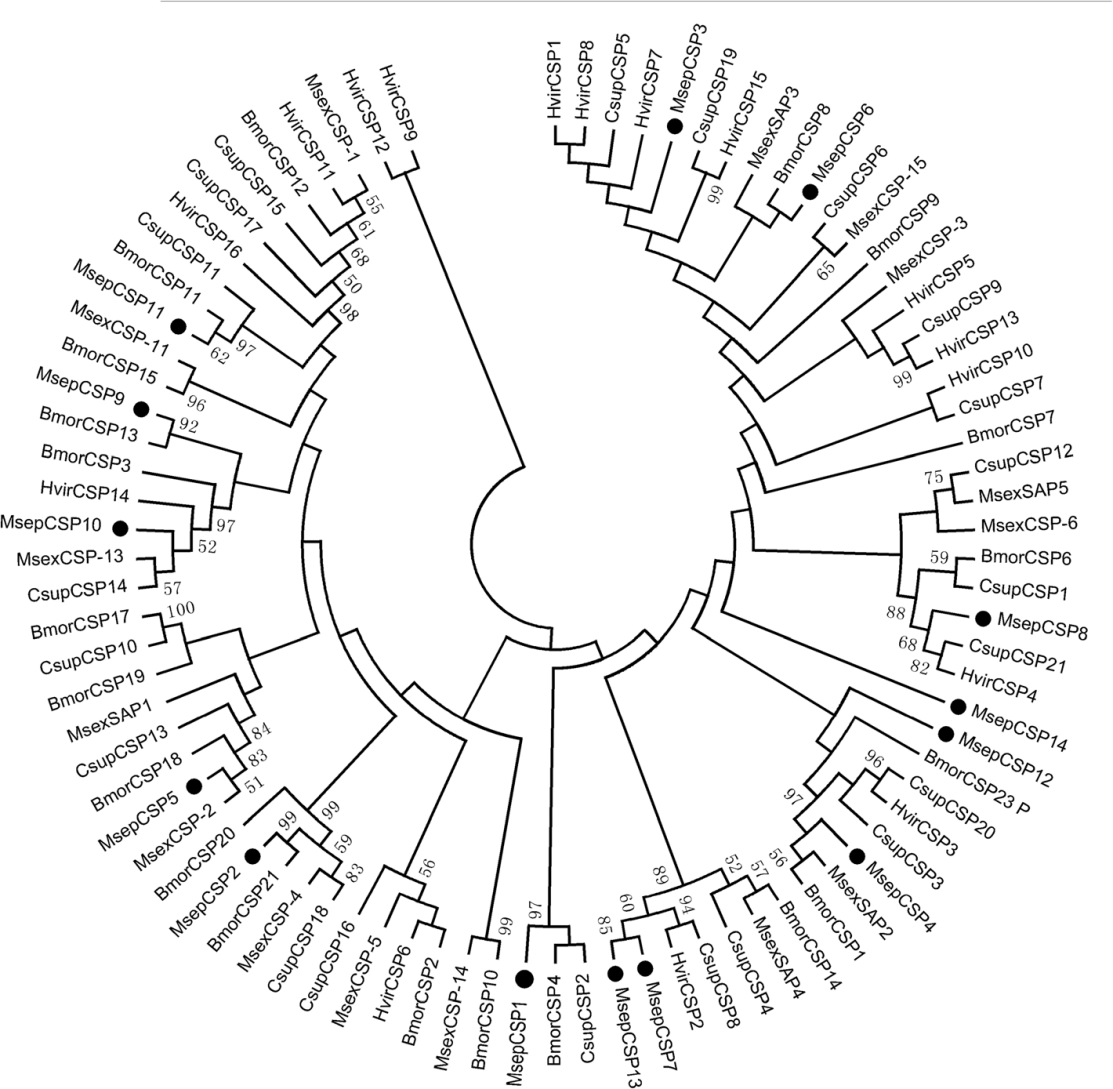
Aligned putative CSP gene sequences of *M. separata* (black circle). Bootstrap values < 50% were ignored. Msep, *M. separata*, Bmor, *B. mori*, Hvir, *Heliothis virescens*, Csup, *C. suppressalis*

**Fig. 8:**
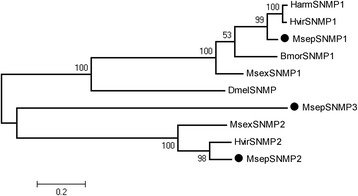
Aligned putative SNMP gene sequences of *M. separata* (black circle). Bootstrap values < 50% were ignored. Msep, *M. separata*, Dmel, *D. melanogaster*, Bmor, *B. mori*, Msex, *M. sexta*, Hvir, *Heliothis virescens*, Harm, *Helicoverpa armigera*

For the 14 CSP genes, 11 CSPs were with full ORF and 4 conserved cysteines were detected in these genes (Table 5, Additional file 2: Figure S3). Except 4 CSPs (CSP3, 6, 12, and 14), all others were clustered with orthologs of other moths in the phylogenetic analysis (bootstrap values > 80) (Fig. 7). For the SNMP genes, 2 genes harbored full ORFs (Table 6). SNMP1 and SNMP2 were clustered with orthologous genes, with bootstrap values > 50, but SNMP3 was not clustered with the other SNMPs (Fig. 8).

**Table 5:** BLASTP results of candidate chemosensory proteins of *M. separata*

Gene name	Full ORF	Protein length (amino acids)	Reference gene name	Reference gene ID	E_value	Similarity (%)
*CSP1*	Yes	149	Chemosensory protein [*Sesamia inferens*]	AGY49270	2.19E-87	87.2
*CSP10*	Yes	122	Chemosensory protein 10 [*Helicoverpa armigera*]	AFR92094	2.62E-76	90.2
*CSP11*	Yes	124	Chemosensory protein [*Helicoverpa armigera*]	AIW65100	1.53E-69	79.0
*CSP12*	No	124	Chemosensory protein [*Batocera horsfieldi*]	AEC04842	1.36E-53	68.5
*CSP13*	No	81	Chemosensory protein 6 [*Agrotis ipsilon*]	AGR39576	2.73E-40	81.5
*CSP14*	No	69	Chemosensory protein 1 [*Delia antiqua*]	BAI82449	6.13E-26	65.2
*CSP2*	Yes	106	Chemosensory protein 8 [*Spodoptera exigua*]	AKT26485	4.54E-60	85.8
*CSP3*	Yes	128	Chemosensory protein 3 [*Agrotis ipsilon*]	AGR39573	3.39E-77	87.5
*CSP4*	Yes	128	Chemosensory protein [*Mamestra brassicae*]	AAF71289	1.8E-70	80.5
*CSP5*	Yes	122	Chemosensory protein 16 [*Spodoptera exigua*]	AKT26491	1.03E-67	80.3
*CSP6*	Yes	127	Chemosensory protein [*Sesamia inferens*]	AGY49267	7.43E-72	79.5
*CSP7*	Yes	127	Chemosensory protein 6 [*Agrotis ipsilon*]	AGR39576	4.09E-84	96.1
*CSP8*	Yes	127	Chemosensory protein CSP3 [*Spodoptera litura*]	ALJ30214	8.88E-68	74.8
*CSP9*	Yes	125	Chemosensory protein 12 [*Spodoptera exigua*]	AKT26488	2.35E-58	71.2
*CSP1*	Yes	149	Chemosensory protein [*Sesamia inferens*]	AGY49270	2.19E-87	87.2
*CSP10*	Yes	122	Chemosensory protein 10 [*Helicoverpa armigera*]	AFR92094	2.62E-76	90.2
*CSP11*	Yes	124	Chemosensory protein [*Helicoverpa armigera*]	AIW65100	1.53E-69	79.0
*CSP12*	No	124	Chemosensory protein [*Batocera horsfieldi*]	AEC04842	1.36E-53	68.5
*CSP13*	No	81	Chemosensory protein 6 [*Agrotis ipsilon*]	AGR39576	2.73E-40	81.5
*CSP14*	No	69	Chemosensory protein 1 [*Delia antiqua*]	BAI82449	6.13E-26	65.2

**Table 6:** BLASTP results of candidate SNMP genes of *M. separata*

Gene name	Full ORF	Protein length (amino acids)	Reference gene name	Reference gene ID	E_value	Similarity (%)
*SNMP1*	Yes	525	Sensory neuron membrane protein [*Mamestra brassicae*]	Q8I9S2	0	94.9
*SNMP2*	Yes	520	Sensory neuron membrane protein-2 [*Heliothis virescens*]	B2RFN2	0	84.2
*SNMP3*	No	184	Sensory neuron membrane protein 3 [*Spodoptera litura*]	AKT26506	2.22E-61	53.8

### RNA expression of olfactory genes in the antennae

**Fig. 9:**
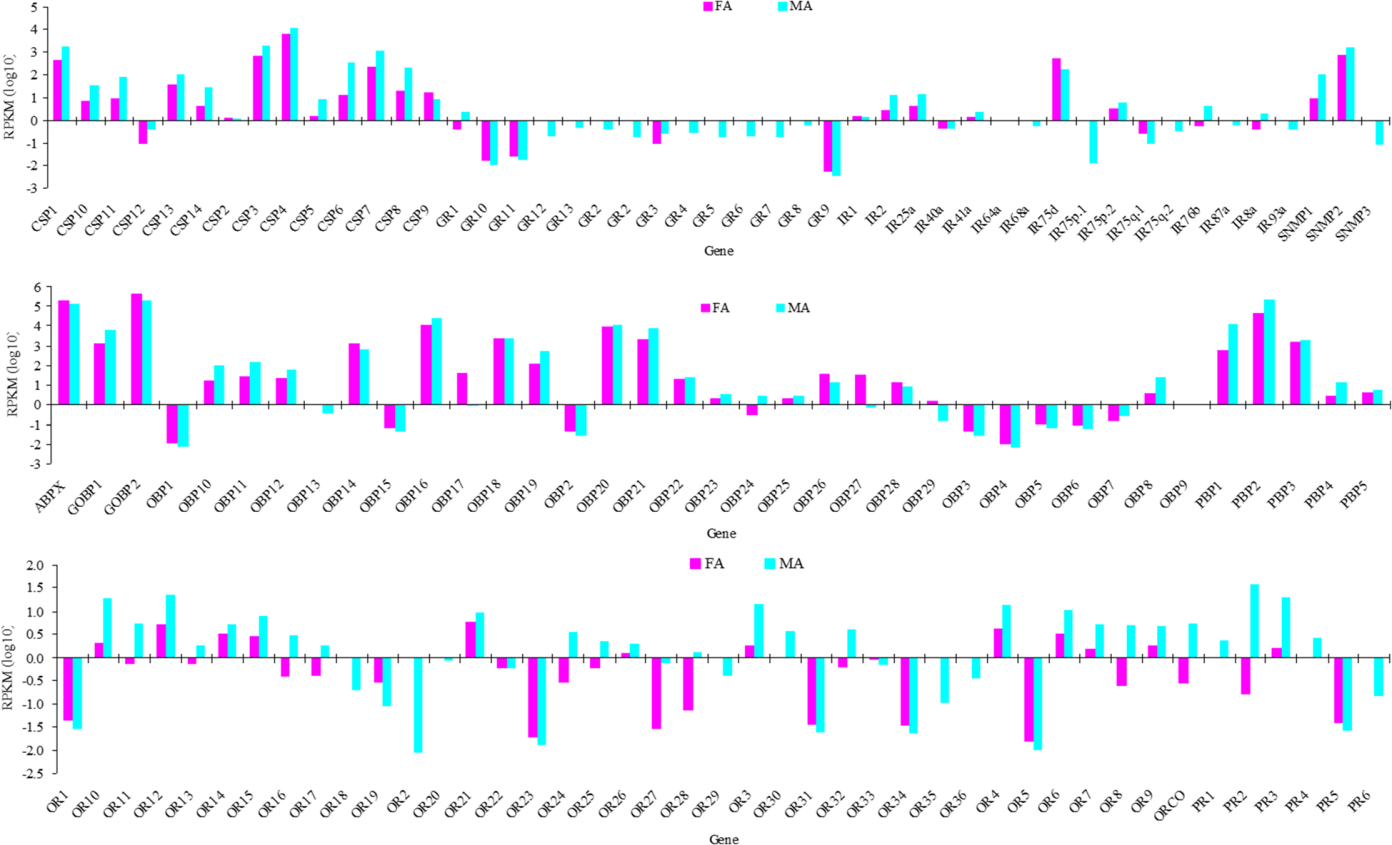
Expression levels of olfactory genes in male and female *M. separata* antennae measured by RNA-Seq. Expression was calculated with log scale of RPKM value

In receptor genes, except for *IR75d* (509.1), the RPKM value of the other receptor genes were < 40 (Fig. 9). The RPKM values of 2 PRs (*PR2* and *PR3*) and 8 ORs (*OR3*, *OR4*, *OR6*, *OR10*, *OR11*, *OR12*, *OR15*, and *OR21*) were larger than that in *ORco* (5.5), and the RPKM values of *IR41a*, *IR76b*, *IR75p2*, and *IR2* were larger than that of *IR8a* (1.9, co-receptor gene of IRs) but smaller than that of *IR25a* (13.2, co-receptor gene of IRs). The RPKM value of *PR2* (38.8) was larger than that of other ORs, and the RPKM value of *PR3* (19.9) was the same as those of *OR12* and *OR10*. The RPKM values of 2 PRs (*PR5* and *PR6*), 15ORs (*OR1*, *OR18*, *OR19*, *OR2*, *OR20*, *OR22*, *OR23*, *OR27*, *OR29*, *OR31*, *OR33*, *OR34*, *OR35*, *OR36*, and *OR5*), and 8 IRs (*IR40a*, *IR64a*, *IR68a*, *IR75p.1*, *IR75q.1*, *IR75q.2*, *IR87a*, and *IR93a*) were < 1 (Fig. 9). Except for *GR1* (2.3), the RPKM values of the gustatory receptor genes were < 0.6.

In contrast to the receptor genes, 16 OBPs and 8 CSPs had RPKM values > 40 (Fig. 9). The RPKM value of *PBP2*, 3 OBPs (*GOBP2*, *ABPX*, and *OBP16*), and *CSP4* were > 10,000, and 2 PBPs (*PBP1* and *PBP3*), 4 OBPs (*GOBP1*, *OBP14*, *OBP18*, and *OBP21*), and 3 CSPs (*CSP1*, *CSP3*, and *CSP7*) were > 1000. The RPKM values of 2 CSPs (*CSP12* and *CSP2*) and 14 OBPs (*OBP1*, *OBP13*, *OBP15*, *OBP2*, *OBP23*, *OBP24*, *OBP25*, *OBP29*, *OBP3*, *OBP4*, *OBP5*, *OBP6*, *OBP7*, and *OBP9*) were < 4. The RPKM value of *SNMP2* was > 1500 (Fig. 9), but the RPKM value of *SNMP3* was very small (0.08).

### Expression of olfactory genes between male and female antennae

**Fig. 10:**
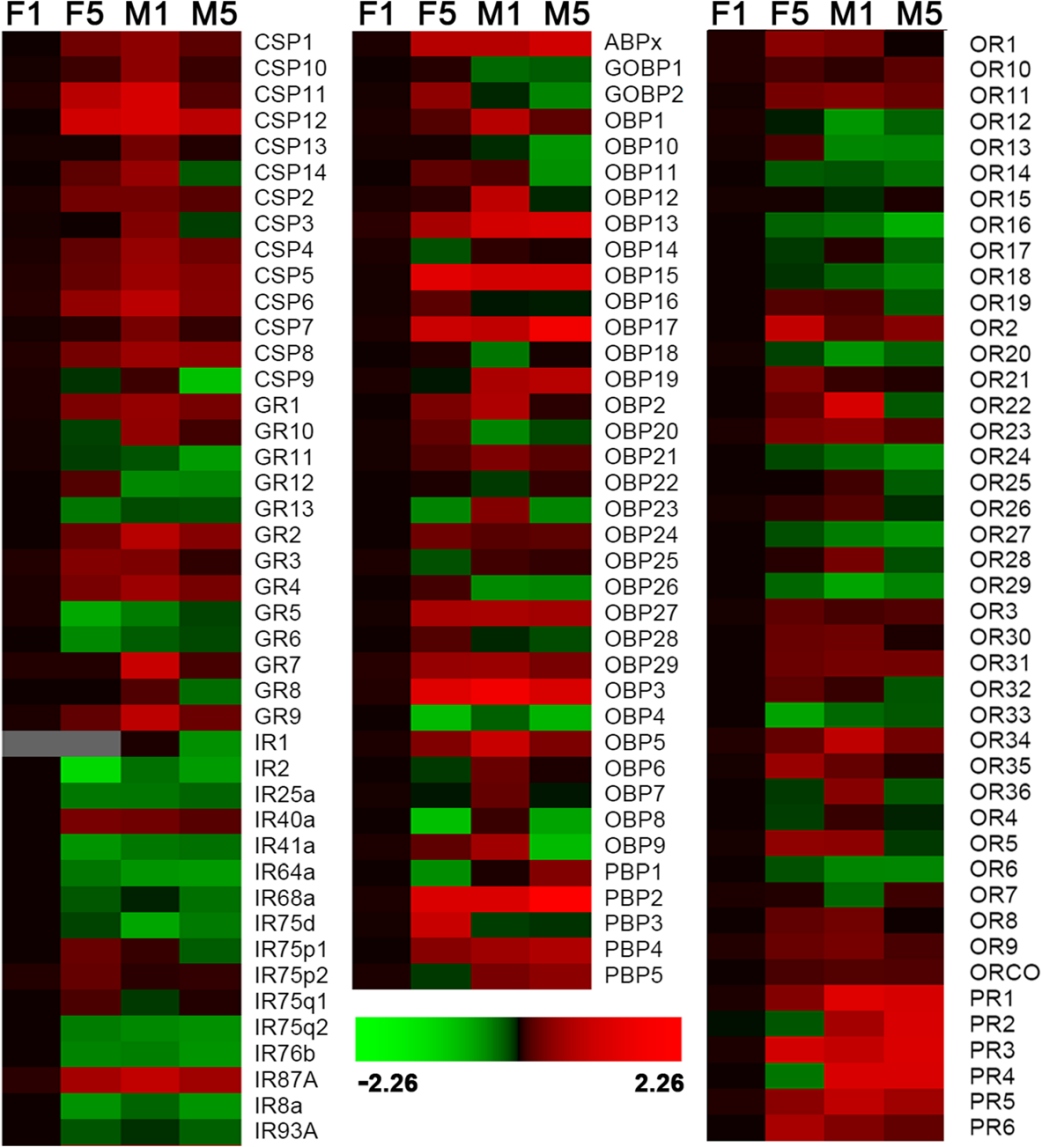
Expression levels of olfactory genes in male and female antennae with different day time measured by RT-qPCR. Gene expression was calculated relative to the *Actin* and *AK* as reference genes and expression in 1-day-old female antennae was arbitrarily defined as control for all genes. Gene expression in other tissue were normalized to 1-day-old female antennae. Log scale of gene expression was used to generate heatmap. F1, 1-day-old female, F5, 5-day-old female, M1, 1-day-old male, M5, 5-day-old male

The expression levels of 43 olfactory genes, including 10 CSPs, 6 GRs, 2 IRs, 16 OBPs and 9 ORs in male antennae were significantly higher than that in female antennae, and the difference among 3 OBPs (*OBP13*, *OBP15*, and *OBP3*) and *IR1* was > 10-fold (Fig. 10). The expression level of 38 olfactory genes, including *CSP9*, 4 GRs, 8 IRs, 9 OBPs and 16 ORs in female antennae was significantly higher than that in male antennae and the difference in *OBP9* was higher by 10-fold. The expression level of 4 IRs, 4 OBPs and 6 ORs was the same between male and female antennae. The difference in expression levels of 16 olfactory genes, including 3 GRs, *IR2*, 3 CSPs, 3 OBPs, and 6 ORs between male and female antennae was influenced by their pre-mating status (Fig. 10).

The expression levels of 4 PBPs (except for *PBP3*) and 5 PRs (except for *PR6*) were significantly higher in male antennae than that in female antennae, and the difference between *PBP2* and the 3 PRs (*PR1*, *PR2*, and *PR4*) was 10-fold higher. The expression level of *PBP3* in 5-day-old females was 10-fold higher than that in males. The expression level of *PR6* in 1-day-old males was significantly higher than that in females, but that in 5-day-old males was significantly smaller than in the female counterpart (Fig. 10).

### Expression of olfactory genes in different pre-mating status

The expression levels of 26 olfactory genes, including 3 CSPs, 2 GRs (*GR1*), 3 IRs, 9 OBPs (*ABPx*, *OBP18*), and 9 ORs (*OR12*, *OR3*, and *OR21*) in 5-day-old moths were significantly higher than that observed in 1-day-old moths, and the difference of *OBP15* was 10-fold higher (Fig. 10). The expression levels of 43 olfactory genes, including 6 CSPs (*CSP3*, *CSP4*, and *CSP7*), 9 IRs (*IR25a* and *IR2*), 7 GRs, 8 OBPs (*OBP14*), and 13 ORs (*OR6*) in 5-day-old moths were significantly smaller than the 1-day-old moths, and difference among *CSP9*, *OBP9*, and *OR22* was 10-fold higher. The expression levels of 14 olfactory genes, including *GR12*, 8 OBPs (*GOBP1*, *OBP16*, and *OBP21*), and 5 ORs (*OR10*, *OR4*, and *ORco*) in 1-day-old moths were similar to those of 5-day-old moths. The differential expression of the 28 olfactory genes, including 5 CSPs (*CSP1*), 3 GRs, 3 IRs (*IR75d*), 7 OBPs (*GOBP2*), and 10 ORs between different pre-mating statuses was influenced by gender, particularly for *CSP11*, *CSP12*, and *OBP3* (Fig. 10).

Three PBPs (*PBP2*, *PBP3*, and *PBP4*) and four PRs (except for *PR4* and *PR5*) were upregulated in 5-day-old moths relative to that in 1-day-old moths, and a 10-fold difference in expression between *PBP2* and *PR3* was observed (Fig. 10). The expression level of *PR4* in 1-day-old females was significantly higher than that in 5-day-old females. The expression level of *PBP5* was the same between 1-day-old and 5-day-old moths. The difference in the expression level of *PBP1* and *PR5* between different pre-mating statuses was influenced by gender.

### EAG responses of antennae to sex pheromones of sympatric species

**Fig. 11:**
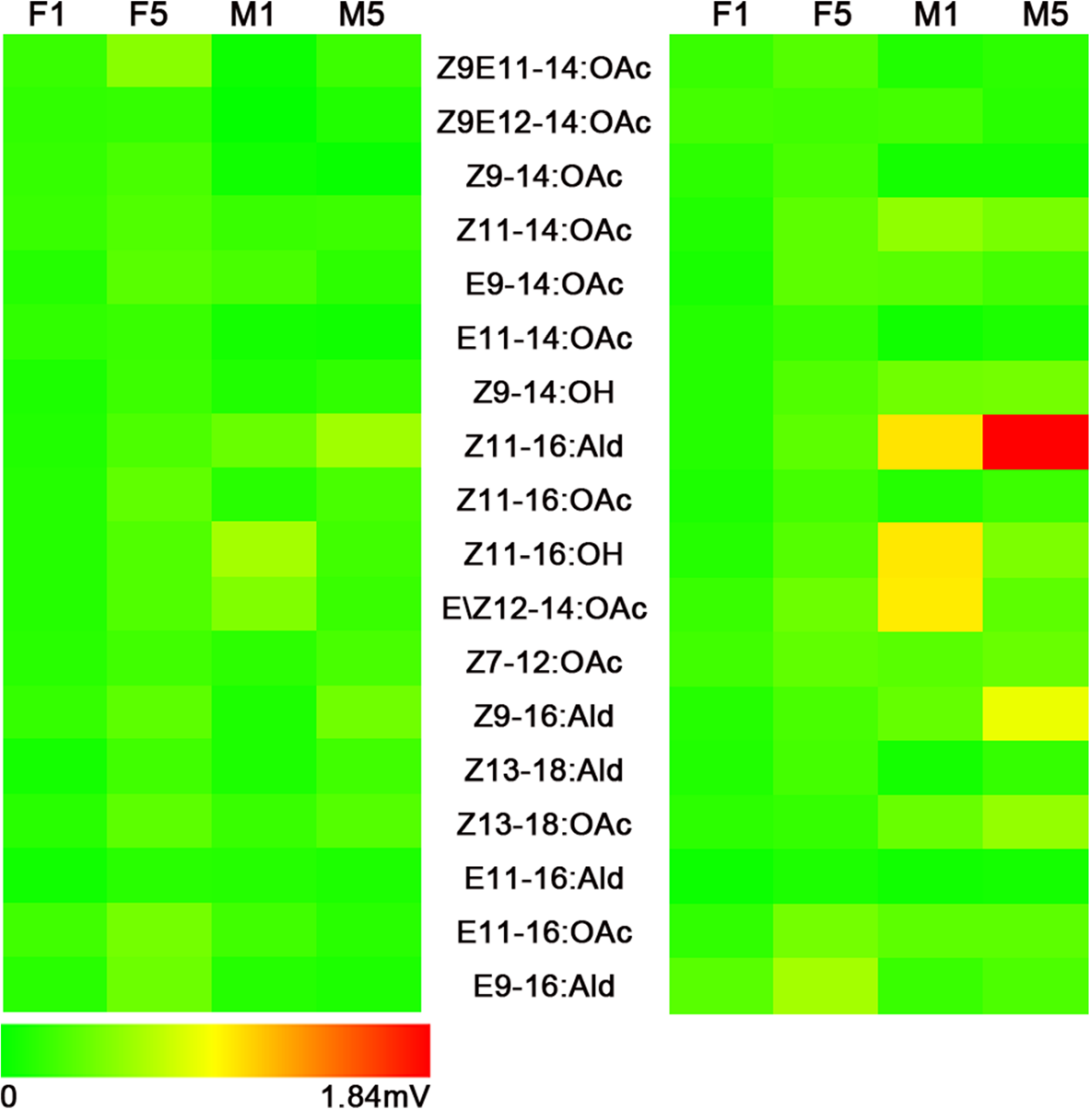
EAG response of male and female moth with different day time to sex pheromone with 10^−4^ (left) and 10^−2^ (right) concentration. F1, 1-day-old female, F5, 5-day-old female, M1, 1-day-old male, M5, 5-day-old male

Antennal EAG response to Z11-16:Ald (1.84 mV) was the strongest in the 18 sex pheromone chemicals, whereas the EAG responses to 11 sex pheromone chemicals of sympatric species (except for Z11-16:Ald, Z9-16:Ald, Z13-18:OAc, Z11-16:OH, E\Z12-14:OAc, Z9E11-14:OAc and (Z)-11-tetradecenyl acetate) were relatively very small (<0.4 mV) (Fig. 11).

In 6 of 7 sex pheromones (except Z9E11-14:OAc) with evident EAG responses (>0.4 mV), the male responses were significantly greater than that in females, and the responses to the 10^−2^ concentration were significantly larger than that observed with the 10^−4^ concentration (Fig. 11). The EAG responses of 5-day-old males to Z11-16:Ald, Z9-16:Ald, and Z13-18:OAc were significantly greater than 1-day-old males, but the EAG responses of 5-day-old males to Z11-16:OH and E\Z12-14:OAc were significantly smaller than 1-day-old males. Female EAG responses to Z9E11-14:OAc were significantly greater than males, and the EAG responses of 5-day-old females were significantly larger than 1-day-old females (Fig. 11).

### EAG responses of antennae to plant odorants

**Fig. 12:**
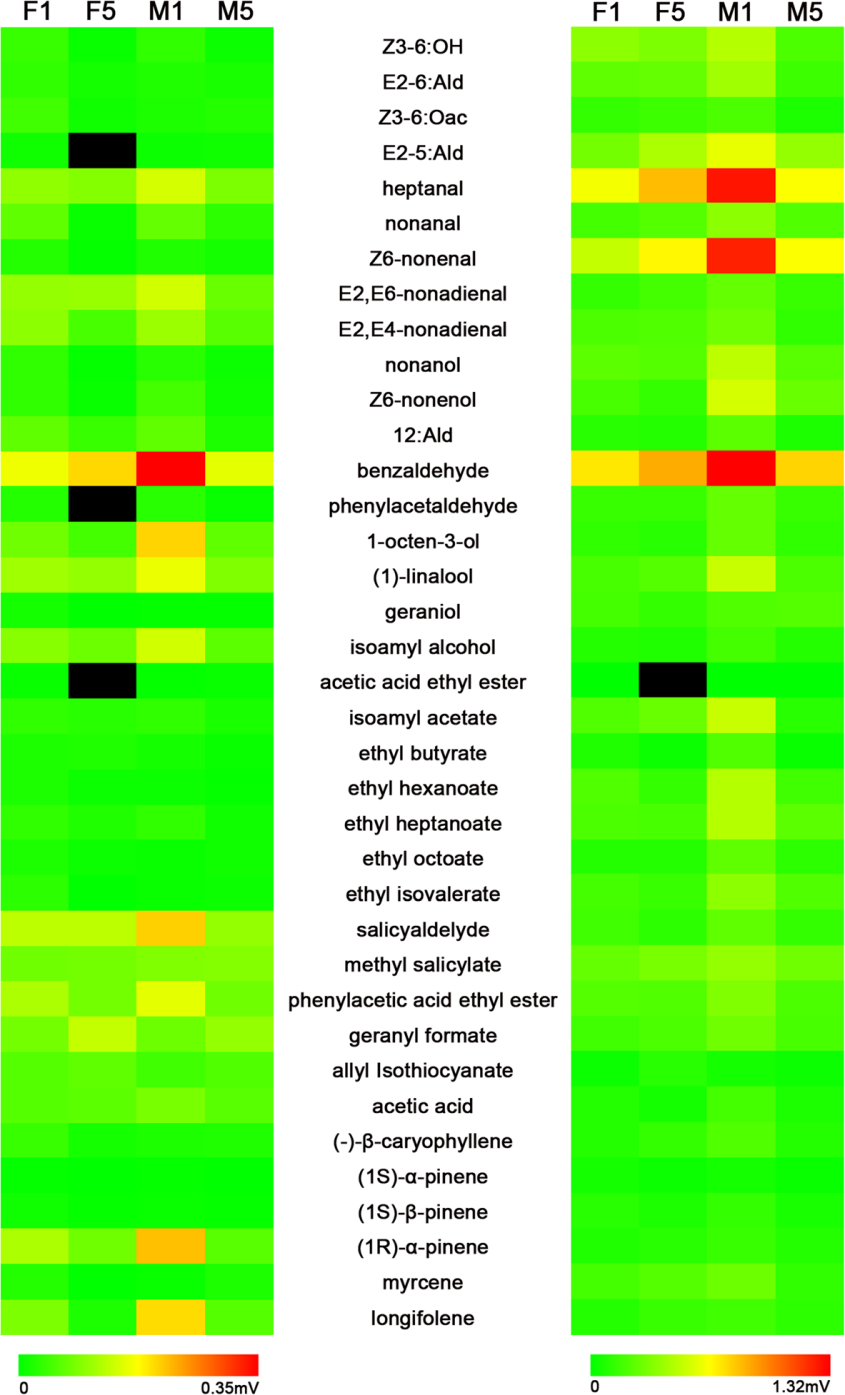
EAG response of male and female moth with different day time to plant volatile with 10^−4^ (left) and 10^−2^ (right) concentration. F1, 1-day-old female, F5, 5-day-old female, M1, 1-day-old male, M5, 5-day-old male

The EAG responses to heptanal, Z6-nonenal, and benzaldehyde were larger than 1.2 mV, but the EAG responses to 21 plant odorants (except for (Z)-3-Hexen-1-ol, (E)-2-Hexenal, E2-5:Ald, heptanal, nonanal, Z6-nonenal, nonanol, Z6-nonenol, benzaldehyde, (1)-linalool, isoamyl acetate, ethyl hexanoate, ethyl heptanoate, ethyl isovalerate, methyl salicylate, and phenylacetic acid ethyl ester) were relatively very small (<0.4 mV) (Fig. 12).

The EAG responses at 10^−2^ concentration were significantly greater than that using 10^−4^. Furthermore, the EAG responses of males were significantly greater than that in females, and 1-day-old males were significantly greater than that in 5-day-old males. At the same time, the EAG responses of 5-day-old females to E2-5:Ald and heptanal were significantly larger than that in 1-day-old females at 10^−2^ concentration (Fig. 12).

## Discussion

### Olfactory genes of *M. separata*

We identified a total of 126 putative olfactory genes in the antennae of *M. separata*, including 43 OR genes, 13 GR genes, 16 IR genes, 37 OBPs, 14 CSPs, and 3 SNMPs. The number of ORs, IRs, OBPs, and CSPs in *M. separate* is smaller than that in *B. mori* [19, 45], which was conducted using the whole genome, but was the same as that of other moths that were studied using the same protocol (antennal transcriptome), which included *Agrotis ipsilon* [46], *C. suppressalis* [37], *H. armigera* [47], *O. furnacalis* [35, 36]. However, the number of GRs and SNMPs was larger than those of most moths studied.

The maxillary palps, which harbor considerably fewer sensilla than antennae, are believed to specialize in taste reception in various moth species [48]. However, 14 gustatory receptor genes were identified in *M. separate* antennae, indicating that the antennae might be an important taste organ for *M. separata*. As shown in a former study, sensilla chaetica were distributed around each antennal segment of *M. separata* [14], which was with a terminal pore and with a shape that was higher and wider than that of other sensilla and have been suggested to have contact/chemoreceptor functions [49]. Sugar as the supplementary nutrient is essential for egg-ripening [8] and as the energy supplement influences the duration of migration [9]. Five sweet receptors were identified in *M. separata* that specifically recognize glucose, galactose, fructose, mannose, sucrose, maltose, trehalose, raffinose, glycerol, and mannitol [50]. GR1, a sweet receptor with higher RPKM than other GRs might be used to recognize the most important sugar for *M. separata*. In addition, the expressions of GR1 in 1-day-old males were larger than that in females and 5-day-old males. Two conserved members of SNMP (SNMP1 and SNMP2) were detected in various moth species [51]. A new SNMP member SNMP3 that has been identified in *S. exigua* recently and other four moths [52] was also reported in *M. separata*. SNMP3 shares only less than 30% identity with SNMP1 and 2 [52].

Different insects have evolved various feeding behaviors in host plants and the evolution of olfactory acuity and discriminatory power of insect must be consistent with the difference in plant odor [53]. Approximately 5 ORs, 2 GRs, 2 IRs, 7 OBPs, and 4 CSPs of *M. separata* were not effectively clustered with those of Lepidoptera (bootstrap values <50). These olfactory genes might be correlated with the special habitat of *M. separata*, including attacking rice, maize, sorghum, wheat, and other gramineous plants. *M. separata* larvae have evolved a specific detoxification system for benzoxazinoid, which is part of the chemical defense system of graminaceous plants [54].

### Co-receptor for odorant detection in *M. separata*

IRs act in combinations of up to three subunits, comprising individual odor-specific receptors and one or two broadly expressed co-receptors, and heteromeric IR complex formation is necessary and sufficient for mediating odor-evoked electrophysiological responses *in vivo* and *in vitro* [55]. IR25a and IR8a function as co-receptors because of their higher sequence identity compared to the other IRs [32], expression in all or most OSN cell bodies, and co-expression with divergent IRs in former studies [56–59]. However, in the present study, the expression levels of *IR8a* (RPKM = 1.9) and *IR25a* (RPKM = 13.2) were evidently smaller than that of *IR75d* (RPKM = 509.1). IR75d has been identified in various insects such as *Grapholita molesta* [60], *Athetis dissimilis* [61], *Rhynchophorus ferrugineus* [62], *B. mori*, *Acyrthosiphon pisum*, *Pediculus humanus humanus*, and dipterans [32]. In *Heliconius melpomene*, the *IR75d* gene is highly expressed across various tissues and sexes [63]. In *D. melanogaster*, IR75d is expressed in three different OSNs that are housed in three coeloconic sensilla (ac1, ac2, and ac4) [64] and is the only ORN class that occurs in more than one sensilla subtype [65]. In addition, IR75d is co-expressed with IR84a and IR76a in ac4 sensilla [66]. These findings suggest that IR75d might be a co-expressed receptor in *M. separate*.

Orco is the common olfactory receptor co-receptor in insects and is present in apparently all OR-expressing ORNs [67]. The expression level of Orco is always higher than that of other ORs [23]. It has been proposed that Orco is a membrane localization protein which stabilizes ORs in the dendritic membranes, as well as a chaperon molecule that facilitates in the correct protein folding of ORs and in forming heteromers with the particular OR as a cation channel [68, 69]. Downregulation of the *Orco* transcript not only results in weaker EAG responses [58, 70–72], but also diminishes the significant behavior preference [72–74] for host volatiles and pheromones. However, the RPKM value of *ORco* (5.5) in *M. separata* was less than 9 general ORs and 2 PRs. The low expression level of ORco in *M. separata* provides allows odorant receptors to mediate odorant responses also in the absence of Orco. When ORs from different species were expressed without Orco in heterologous expression assays such as *Drosophila* Schneider 2 (S2) and *S. frugiperda* 9 (SF9) cells, these also evoked ligand-specific responses [75]. In addition, the functional expression of BmOR-1 and BmOR-3 in modified HEK 293 cells was possible without co-receptor BmOR-2 and BmOR-3 being heterologously expressed. Furthermore, modified HEK 293 cells are highly sensitive and selective responsiveness to bombykal [76]. The Orco agonist VUAA1 did not collectively alter mating efficiency, the number of eggs produced, and the number of hatched nymphs in the bed bug, *Cimex lectularius* L [77]. In fact, Orco is not essential for odorant detection when ORs are successfully inserted into the plasma membrane [75].

### Pheromone recognition of *M. separata*

The sex pheromone of *M. separata* isolated from Japan and Taiwan was identified as a blend of (Z)-11-hexadecenyl acetate (Z11-16:Ac) and (Z)-11-hexadecenol (Z11-16:OH) [78, 79]. However, Z11-16Ald is the main female sex pheromone in mainland China [10, 11], and male moths had the highest peak response to Z11-16:Ald [15]. In the present study, the EAG response of male oriental armyworm to Z11-16:Ald was significantly greater than that to other pheromones. In addition, the male EAG response to Z11-16:Ald was significantly greater than that in females, and the EAG response to the 10^−2^ concentration was significantly greater than that to the 10^−4^ concentration. However, the EAG responses to Z11-16:Ac was very small (<0.2 mV) and were not significantly influenced by concentration, sex, and pre-mating status. Z11-16:Ald is the main female sex pheromone in *M. separata*. The calling behavior of females is the most frequent and the sex pheromone titer in the sex gland is the highest in 4–5 day old *M. separata* [80]. In the present study, the EAG responses to Z11-16:Ald of 5-day-old males were significantly greater than that of 1-day-old males, which was consistent with the female calling period of *M. separata*. In all candidate genes for sex pheromone recognition, *PR2* and *PBP2* were the most abundant PR and PBP, respectively, and were specifically expressed in male antennae. In addition, the expression level of *PR2* and *PBP2* in 5-day-old males was 10-fold higher than those found in 1-day-old males. The consistency between the EAG response to Z11-16:Ald and the expression profile of *PR2* and *PBP2* suggests that these two genes might be used in recognizing Z11-16:Ald, the main female sex pheromone.

In addition to Z11-16:Ald, there were also EAG responses to other sex pheromones such as Z9-16:Ald, Z11-16:OH, and E\Z12-14:OAc. Male EAG responses to these pheromones were significantly greater than female EAG responses, and the responses to the 10^−2^ concentration were significantly greater than that to the 10^−4^ concentration. These sex pheromones might be identified by male *M. separata*. Besides PR2 and PBP2, there were also other 5 PRs and 4 PBPs in the antennae and the expression levels of 3 PBPs and 4 PRs in male antennae were significantly higher than the females. These genes might be used in recognizing former sex pheromones. For example, *MsepPR1* is used as a specific pheromone receptor for Z11-16:Ac, which is the main sex pheromone component of the Japanese *M. separata* [24]. EAG responses of 5-day-old males to Z9-16:Ald was significantly greater than that of 1-day-old males in the present study, and Z9-16:Ald might be a minor sex pheromone component in *M. separata* female glands [15]. In accordance with the EAG response, the expression levels of 2 PBPs (*PBP1* and *PBP3*) and *PR3* were higher in 5-day-old male moths than in the 1-day-old moths. E\Z12-14:OAc is the main female sex pheromone of *O. furnacalis*, which is a major pest of maize [81]. *M. separata* males might be capable of distinguishing conspecific females from other species by recognizing their sex pheromone components or might search host plants with female sex pheromone of moths that infest the same host as that of *M. separata*. For example, *M. separata* might find maize plants by recognizing E\Z12-14:OAc.

### Host recognition in *M. separata*

At least four distinct large-scale and long-distance migration events of *M. separata* between overwintering sites in southern China and northern temperate zones occur annually, including two northward displacements in the spring and early summer and two southward displacements in summer and fall [3]. The migration takes place in 1–2 days after eclosion for the orient armyworm [5]. Adults need a sufficient amount of sugar as the energy supplement for flight, and sugar is a crucial factor that influences the duration of migration [9]. In addition, adults also need a sufficient amount of sugar for survival; otherwise, these soon perish [8]. Under natural conditions, the adult armyworm feeds on flower nectar or honey-dew of aphids [50]. In the present study, EAG responses of 1-day-old moths to 16 plant volatiles were significantly larger than the responses of 5-day-old moths, and expression level of the 43 olfactory genes (6 CSPs, 9 IRs, 7 GRs, 8 OBPs, and 13 ORs) in 1-day-old moths were significantly higher than those in the 5-day-old moths. These plant volatiles might be used by an adult armyworm to search for food, and might be mediated by these olfactory genes, which were differentially expressed in 1- and 5-day-old moth antennae. In particular, the EAG responses to heptanal, Z6-nonenal, and benzaldehyde were greater than 1.2 mV, which is the EAG response to sex pheromones. Heptanal, Z6-nonenal, and benzaldehyde are flower volatile compounds [82, 83]. These three flower volatiles might be very important to adult armyworms as they search for food. The RPKM values of the 7 OBPs and 4 CSPs were > 1000, whereas that of the 6 ORs and 2 IRs were > 10. Therefore, these olfactory genes might play a critical role in the recognition of host volatiles.

Spermatids are observed at the time of male *M. separate* emergence [8], whereas oocytes ripen only after the female moth has consumed a sufficient amount of food [84]. In accordance with the larger sugar requirement of the female, the expression levels of 38 olfactory genes (*CSP9*, 4 GRs, 8 IRs, 9 OBPs, and 16 ORs) in females were significantly higher than those in the males (Fig. 10). However, no plant volatiles with higher female EAG responses than that in the males were observed in the present study. The inconsistency between gene expression and EAG response might be attributable to the loss of important chemicals. In fact, identifying all stimulating chemicals may be a relatively difficult task. A panel of 480 odorants, including esters, acids, aldehydes, ketones, alcohols, pyrazines, aromatics, terpenes, and sulfur compounds, against 21 larval odorant receptors (more than 10,000 odorant–receptor combinations) were conducted in fly, but no strong odorants were identified for *Or2a* and *Or49a* [85]. Other than sex pheromones, host plant volatiles might also be used by sexually mature males to find females for mating. In Lepidoptera, several cases of synergism between plant semiochemicals and pheromones have been observed both in the laboratory and field [86]. In our study, the male responses to 16 plant volatiles with high EAG responses (>0.4 mV) were significantly greater than the female responses, and the expression levels of 43 olfactory genes (10 CSPs, 6 GRs, 2 IRs, 16 OBPs, and 9 ORs) in males were significantly greater than in the females. These results prove that some plant volatiles are very important to male moths, and using of these volatiles might be a strategy to optimize mating opportunities. However, our understanding of how insect responses to plant stimuli aid in mating are limited [87].

In the present study, the EAG response to volatile organic compounds of larvae host plants was very small, which include β-myrcene, (Z)-3-hexenyl acetate, nonanal, linalool, β-caryophyllene, (E)-2-hexenal, and (Z)-3-hexen-1-ol from wheat [88, 89]. These low EAG responses may be attributable to the pre-mating status of the moths used in the present study. Only 1- and 5-day-old unmated male and female moths were used in our study. The most important behavior of 1- and 5-day-old moths involved finding their nutrition for sex maturation as well as searching for a mating partner. Therefore, volatiles of larvae host plant might not be required by 1- and 5-day-old unmated moths.

## Additional files

Additional file 1: Table S1. Chemicals tested in the Electroantennogram recordings. **Table S2.** Primers used in the study. (DOC 267 kb)
Additional file 2: Figure S1. Size distribution of all 41,056 unigenes assembled from the pooled *M. separata* RNA extract. **Figure S2.** Aligned putative full ORF of OBP gene sequences of *M. separata*. Six conserved cysteines were highlighted by blue color. **Figure S3.** Aligned putative ORF of CSP gene sequences of *M. separata*, CSP13 and CSP14 excluded. Four conserved cysteines were highlighted by blue color. (DOC 683 kb)

## Abbreviations

ABPX: Antennal binding protein X
AK: Arginine kinase
CSPs: Chemosensory proteins
E\Z12-14:OAc: (E)/(Z)-12-tetradecenyl acetate
E2-5:Ald: (E)-2-Pentenal
EAG: Electroantennogram
eggNOGe: Volutionary genealogy of genes: Non-supervised Orthologous Groups
GOBPs: General odorant binding proteins
GRs: Gustatory receptors
IRs: Ionotropic receptors
OBPs: Odorant binding proteins
ORco: Olfactory receptor co-receptor
ORs: Olfactory receptors
PBPs: Pheromone binding proteins
PR: Pheromone receptor
RPKM: Reads per kb per million reads
RT-qPCR: Quantitative reverse transcription PCR
SNMPs: Sensory neuron membrane proteins
Z11-16:Ald: (Z)-11-Hexadecenal
Z11-16:OH: (Z)-11-hexadecenol
Z13-18:OAc: (Z)-13-Octadecenyl acetate
Z9-16:Ald: (Z)-9-Hexadecenal
Z9E11-14:OAc: (9Z,11E)-tetradecadienyl acetate
