# Supplementary material for: Age influences the olfactory profiles of the migratory oriental armyworm mythimna separate at the molecular level
Source: BMC Genomics. 2017 Jan 5;18:32. doi: 10.1186/s12864-016-3427-2 (PMC5217624; doi:10.1186/s12864-016-3427-2)
Supplement: Additional file 1: Table S1. — Chemicals tested in the Electroantennogram recordings. Table S2. Primers used in the study. (DOC 267 kb) [file 12864_2016_3427_MOESM1_ESM.doc]

Table S1 Chemicals tested in the Electroantennogram recordings

|  | **Chemicals** | **Purity** | **Sources** |
| --- | --- | --- | --- |
|  | **Insect pheromone compounds and their isomers** |  |  |
| 1 | (9Z,11E)-tetradecadienyl acetate (Z9E11-14:OAc) | 92% | Bedoukian Research Inc |
| 2 | (9Z,12E)-tetradecadienyl acetate (Z9E12-14:OAc) | 93% | Bedoukian Research Inc |
| 3 | 9Z-tetradecenyl acetate (Z9-14:OAc) | 95% | Bedoukian Research Inc |
| 4 | 11Z-tetradecenyl acetate (Z11-14:OAc) | 95% | Bedoukian Research Inc |
| 5 | 9E-tetradecenyl acetate (E9-14:OAc) | 96% | Bedoukian Research Inc |
| 6 | 11E-tetradecenyl acetate (E11-14:OAc) | 98% | Bedoukian Research Inc |
| 7 | 9Z-tetradecen-1-ol (Z9-14:OH) | 95% | Bedoukian Research Inc |
| 8 | 11Z-hexadecenal (Z11-16:Ald) | 95% | Bedoukian Research Inc |
| 9 | 11Z-hexadecenyl acetate (Z11-16:OAc) | 95% | Bedoukian Research Inc |
| 10 | 11Z-hexadecen-1-ol (Z11-16:OH) | 98% | Bedoukian Research Inc |
| 11 | 12 E\Z -tetradecenyl acetate (E\Z12-14:OAc) |  |  |
| 12 | 7Z-dodecenyl acetate (Z7-12:OAc) |  |  |
| 13 | 9Z-hexadecenal (Z9-16:Ald) |  |  |
| 14 | 13Z-octadecenal (Z13-18:Ald) |  |  |
| 15 | 13Z-octadecennyl acetate (Z13-18:OAc) |  |  |
| 16 | 11E-hexadecenal (E11-16:Ald) |  |  |
| 17 | 11E-hexadecenyl acetate (E11-16:OAc) | 93% | Bedoukian Research Inc |
| 18 | 9E-hexadecenal (E9-16:Ald) | 93% | Bedoukian Research Inc |
|  |  |  |  |
|  | **Green leaf volatiles** |  |  |
| 15 | cis-3-hexen-1-ol (Z3-6:OH) | 98% | Sigma Aldrich Inc. |
| 16 | trans-2-hexenal (E2-6:Ald) | 97% | Sigma Aldrich Inc. |
| 17 | cis-3-hexenyl acetate (Z3-6:OAc) | 99% | Sigma Aldrich Inc. |
| 18 | E2-pentennal (E2-5:Ald) | 95% | Jiaxing Fragrance Inc. |
| 19 | heptanal | 97% | Aladdin Regent database Inc. |
| 20 | nonanal | 97% | Aladdin Regent database Inc. |
| 21 | Z6-nonenal | 97% | Aladdin Regent database Inc. |
| 22 | E2,E6-nonadienal | 93% | Aladdin Regent database Inc. |
| 23 | E2,E4-nonadienal | 93% | Aladdin Regent database Inc. |
| 24 | nonanol | 97% | Aladdin Regent database Inc. |
| 25 | Z6-nonenol | 97% | Aladdin Regent database Inc. |
|  |  |  |  |
|  | **Floral aromatic compounds** |  |  |
| 26 | dodecyl aldehyde (12:Ald) | 97% | Aladdin Regent database Inc. |
| 27 | benzaldehyde | 99% | Sigma Aldrich Inc. |
| 28 | phenylacetaldehyde | 90% | Sigma Aldrich Inc. |
| 29 | benzyl alcohol | 98% | Bodi Chemicals Inc. |
| 30 | 1-octen-3-ol | 97% | Aladdin Regent database Inc. |
| 31 | (1)-linalool | 97% | Sigma Aldrich Inc. |
| 32 | geraniol | 97% | Sigma Aldrich Inc. |
| 33 | isoamyl alcohol | 97% | Aladdin Regent database Inc. |
| 34 | acetic acid ethyl ester | 99% | Aladdin Regent database Inc. |
| 35 | isoamyl acetate | 99% | Aladdin Regent database Inc. |
| 36 | ethyl butyrate | 97% | Aladdin Regent database Inc. |
| 37 | ethyl-2-methyl butyrate | 97% | Aladdin Regent database Inc. |
| 38 | ethyl hexanoate | 97% | Aladdin Regent database Inc. |
| 39 | ethyl heptanoate | 97% | Aladdin Regent database Inc. |
| 40 | ethyl octoate | 99% | Aladdin Regent database Inc. |
| 41 | valeric acid ethyl ester | 98% | Aladdin Regent database Inc. |
| 42 | cis-3-hexenyl butyrate | 97% | Aladdin Regent database Inc. |
| 43 | ethyl isovalerate | 97% | Aladdin Regent database Inc. |
| 44 | salicyaldelyde | 97% | Aladdin Regent database Inc. |
| 45 | methyl salicylate | 98% | Bodi Chemicals Inc. |
| 46 | phenylacetic acid ethyl ester | 99% | Aladdin Regent database Inc. |
| 47 | geranyl formate | 97% | Aladdin Regent database Inc. |
| 48 | allyl Isothiocyanate | 98% | Sigma Aldrich Inc. |
| 49 | γ-unsecalactone | 95% | Jiaxing Fragrance Inc. |
| 50 | acetic acid | 97% | Aladdin Regent database Inc. |
|  |  |  |  |
|  | **Terpenes** |  |  |
| 51 | (-)-β-caryophyllene | 90% | Tokyo Chemical Industry Co., Ltd. |
| 52 | 3-carene | 97% | Sigma Aldrich Inc. |
| 53 | (1S)-α-pinene | 98% | Sigma Aldrich Inc. |
| 54 | (1S)-β-pinene | 98% | Sigma Aldrich Inc. |
| 55 | (1R)-α-pinene | 98% | Sigma Aldrich Inc. |
| 56 | myrcene | 97% | Aladdin Regent database Inc. |
| 57 | longifolene | 97% | Aladdin Regent database Inc. |
| 58 | 1,8-cineole | 95% | Jiaxing Fragrance Inc. |

Table S2 Primers used in the study

| Gene name | Forward primer (5’-3’) | Reverse primer (5’-3’) | Tm (℃) |
| --- | --- | --- | --- |
| *ABPX* | ACATGAAGTGCACCATGGAG | TCTTCCGATCTCAGCCTTGTTG | 60 |
| *CSP1* | ACAATGAACGACTGCTGACC | ATCGATGAGGTAGTGCATCACC | 60 |
| *CSP10* | TTTGGTCGTGTTGGCGTTTG | AGCAGTTGATGTACGCCTTG | 60 |
| *CSP11* | ATCCGCGTTGTTGTCAATGG | TCCTTGAATTCGCCATTGGG | 60 |
| *CSP12* | ACAACGAGTGTGCCAAATGC | TTCCTTCCACCAGTCACTCTTG | 60 |
| *CSP13* | GCGCCTTCTGAAAGCTTATGTC | AAGAGCCTTTCCATCAGGAGTG | 60 |
| *CSP14* | GCAGGCAAGGAACTCAAATCTC | TGCTTGCGTATTTGCCTTCG | 60 |
| *CSP2* | TGCAGCGACACATCAAATGC | TGGACAAATGCAAGGGTTCG | 60 |
| *CSP3* | ACGTCAAGTGCATTCTCGAC | ATTTGGCGCACTCATTCTCG | 60 |
| *CSP4* | AAGTTCGTGCTACTGCTGTG | TCTTTAAGCTCCTTGCCCTCAG | 60 |
| *CSP5* | AACTGCATGCTGGACAAAGG | CATTTGGCGCATGATGTTGC | 60 |
| *CSP6* | TATTCGCACTTGTGGCGTTG | ACACTTGATGTAGGGCACCAG | 60 |
| *CSP7* | TCACAGCGCCTTCTAAAAGC | AAGTCTGGACGCTTGTTCAC | 60 |
| *CSP8* | ACAGGGTGCGCAAAATGTAC | TGCCAGGGTCGTATTTGTTG | 60 |
| *CSP9* | AAAAACTGTTGCCCGAAGCC | ATAGCCTTGGCTACATGCAC | 60 |
| *GOBP1* | ACGACTTCAAGTTCGAGCAC | ATGAACTGCTCGGTGTTGTC | 60 |
| *GOBP2* | TTGGGAAGGCTCTTGAAGAGTG | TTGGACATGCAGATGATGGC | 60 |
| *GR1* | TGCTGTTGTGCTGTTGTTGG | AATGTGTGACGAAGCCTTGG | 60 |
| *GR10* | TGATAGTGTGTTCAGCGGACTG | TCAGAAATCGCACGAGCATG | 60 |
| *GR11* | ATGCTCATTGGTCGCTCTTG | TCATATCGCAAGCACATGCC | 60 |
| *GR12* | TCGCCATTTGGAGAGTTGTG | AAAGTCAAGGGCCGTGTTAG | 60 |
| *GR13* | AGCGATTTGAAGCCAGACAG | GGTTGATTGACGCACGTACG | 60 |
| *GR2* | AAAGCGCGTCACCTCTAAAC | AATCCACCAGTAGGCTCACG | 60 |
| *GR3* | TGCTTGCAGCTGTTTTTGGG | ACTGCACGTTGATGTCAGAG | 60 |
| *GR4* | ACAATGGTGCACTTCAAGCC | GCGCTTTGAAAATCCTCAGC | 60 |
| *GR5* | AATGTTTTCGGAGGGACGTG | TAAGCGTTTCCCAGCGATTG | 60 |
| *GR6* | TGAACCTTGATGGCTTCACC | TCAGCTTGAACTGCATCAGC | 60 |
| *GR7* | ATGCTGTCGTCACAAGATGG | ATCATGATGCGCTCGTAACC | 60 |
| *GR8* | TGCGCACTTGTCTTGATGTC | CGCTGATCTTGCTGAATGACG | 60 |
| *GR9* | TGTCAAGACAGTCACAAGCG | TGAACAGACCACATGCTGTC | 60 |
| *IR1* | TGTTCCTCTTTGCTGTGCTG | TTTTTGTGCCTCGCTCACTG | 60 |
| *IR2* | TCAACAGCTCGTCAAGTCAC | TCAGGGCATTGCGTTTGTTG | 60 |
| *IR25a* | AGGAAAACTGCGTGGTTTGC | TCATGTCGTTTGGCCACTTG | 60 |
| *IR40a* | AAGAACCTTCCCGAACAAGC | TAACTTCGCCGCCTTGTTTG | 60 |
| *IR41a* | TAACGCAAGAAGCCATGCTG | TTGTGGTGCCGACATCATTC | 60 |
| *IR64a* | TTAAGCGCACAACAGATCCG | TGCAGCGGATACATTTGCAC | 60 |
| *IR68a* | ACACTTACGGAATGCTGCTC | TCCGCCACAAGTTACTTTGC | 60 |
| *IR75d* | TCATCAAGCAGACGTTCACG | TCAATGATGCCGATGCTGAC | 60 |
| *IR75p.1* | TCAACTCGCCACTCAAGTTG | ATGTGGAACGCGAACAAACC | 60 |
| *IR75p.2* | TGGCAACAGAACGCAAACTC | AAGCGGTCTCATTTGCAAGC | 60 |
| *IR75q.1* | CTGCTGTATGGAAAGATCTTGGC | TGCTTCTTCTCTTCTGCAGACG | 60 |
| *IR75q.2* | TGTGGACTGCGAGAGATTCAG | TCGGTCGTTTCTCGTACATCAG | 60 |
| *IR76b* | AAGAAACAGAGGGCATTCGC | GCAGCAAAAACAGCAACAGC | 60 |
| *IR87a* | AACTTCGCAGTCGTTTCGTC | ATTAATGGCGCCCCTTTACC | 60 |
| *IR8a* | AAGTGTGGCTCAGCATTGTG | TAAACGACGTCAACGCGAAC | 60 |
| *IR93a* | AGCAGCGCTAACTTGACAAC | ATGTGCGGAAACAGAACGTC | 60 |
| *OBP1* | AGGTGCAAATAGACGCATGC | GAAAACGGACACACTAGAGGAG | 60 |
| *OBP10* | AGCGTGCTTTGAAGAGTTGC | TCAGGCATCGTTCCAACTTG | 60 |
| *OBP11* | ATGCTGCTGACAAACCCAAC | TGTTTGGGGTCTTTCTCGTG | 60 |
| *OBP12* | ACATTTGGGCTGACTCCAAC | ATGCGCTTTCAGAACTCGAC | 60 |
| *OBP13* | AGAACTTGGAGTCCAGAAGCTG | TTGCCTTCATCACGTTGCAC | 60 |
| *OBP14* | TTCTTGCGTTCTGTGTTGCG | TCATACTTGGCGCATTTGGC | 60 |
| *OBP15* | AGACGTCAGGGCATACTTTACC | ATCAGACATGCCTTCGATGC | 60 |
| *OBP16* | TCATCACATTCGCGCTAACC | AGCGGATGAAGCATTTAGGC | 60 |
| *OBP17* | TGTGCATGTCCAAGAAAGCG | AGCAGATTGGCGGTCTTTTG | 60 |
| *OBP18* | TGTACTTGAATGCGCGGATG | TCGCCGTCGAAAAATTCTGC | 60 |
| *OBP19* | AGGCTGCTGTCGATAAATGC | TGGGTTGAACTCGTACATGC | 60 |
| *OBP2* | AGCGTTTATGCAGGAACAGC | TTTTATCGTCCGCGCATTCG | 60 |
| *OBP20* | TGCTGCTGTGTTTAGTTGCC | AACACACTCGTCGTGAACTG | 60 |
| *OBP21* | AAGTTCGTGCCCGATGAAAC | TCCAAGAAGCAAGCTGTCAG | 60 |
| *OBP22* | TGGGTGACATCGAAAAAGGG | AGGCTTCGCACAAATCCTTG | 60 |
| *OBP23* | ACGGAGATGAAAAGCGACTG | AAAGCAGCTCTGTCACAACC | 60 |
| *OBP24* | GCTACGCTGAAACCTTTGTCAG | ACATTTCGCCAGGTGCTTTG | 60 |
| *OBP25* | TTCACGGAAGCGTGCAAAAG | AGATCAAAGCAGCTCTTTCGC | 60 |
| *OBP26* | GACGCTGCCATAAAACAAGC | ATCCGCAGCTTTCTTGCAAG | 60 |
| *OBP27* | ACCAAAGCCAGGAAAGGAGAG | TCCGTCAGCAGTCATGAAAC | 60 |
| *OBP28* | TGAAGACCCGAACATCATGTG | ACGCAACGGCCTTTTTAGTC | 60 |
| *OBP29* | AATTCCGGGCCTTCGTAAAG | TTTCTCTGTGCAGGCAATGG | 60 |
| *OBP3* | TTCTCGCCGCCTAGAAAAAC | CTTCACCGTCGGTTACTGTTTC | 60 |
| *OBP30* | AGAGCAAAATCGGCCTTTCC | ATGTCACGCCGTTTTTGTCC | 60 |
| *OBP31* | ATAGCCACAGCACACACATG | TGTTACCGCCGTCATGAAAC | 60 |
| *OBP32* | TCAAGGCCGAGCTGACTTTG | ATCTACGCCAGTTTGCTTGG | 60 |
| *OBP33* | GCGCCTGTTAACAACGAATC | TACACTTGTACGCCAGCATG | 60 |
| *OBP34* | GCCCTGACCAACCTTAGAAAAC | ACCGCTTGTGCATTGTCTAG | 60 |
| *OBP35* | CTGCAGTGAATCAACAAAAGACG | CGTATTTTGGCGCATTTTCCG | 60 |
| *OBP4* | AGAAGAACGGCACTGAAACG | GTTTGCCAGTTTTCGTTCGC | 60 |
| *OBP5* | CGTAGCTTGCATACTGAAACGG | ACGCGTACTCACAAACTTCG | 60 |
| *OBP6* | ACAGAGGTAGTTGCAGAAGGAC | TAGCATAAGCCGTAGCCTTAGC | 60 |
| *OBP7* | AGAATGCCGCAAAACAACCG | TCTCCACCATCTTCGACGAAAG | 60 |
| *OBP8* | AGCGAAGAAAGGCCACATTG | ACCGGCTTTCTTGAAGAAGC | 60 |
| *OBP9* | TGGCAAAGACGGAAAGATGC | ATGGCTTTTCCGCACAAAGC | 60 |
| *OR1* | ACGGCATTGGTGAAGACAAC | TGCCGGTTTTGTTGTCTCTG | 60 |
| *OR10* | TGCAGCATCGTCGTCAAAAC | AGACGAAGGTAAACCATGCG | 60 |
| *OR11* | ACGGGTGACACTTTCAACAC | TGCCGATGTTGCTTAACACC | 60 |
| *OR12* | TTGTGTAGCGCAGTTGTACG | TTGAATGCTCGCGTGTGTTG | 60 |
| *OR13* | AGCAGCATGCCTTGACTTTG | TGGAAATAAGCAGGCCACAC | 60 |
| *OR14* | ATGTCGGAAAGTGAGATGCG | TCCAAAGCCTTGCAGTACAG | 60 |
| *OR15* | TGTTCGCTGATGCTTTGTGC | ACGAAAAGTTGTGCGACTCC | 60 |
| *OR16* | TTTCAGTTTGGCGTCAGCTC | TCGAATGCGGCTTTTGTGAG | 60 |
| *OR17* | GCGCTGGACCTTTCAATTTC | TCCGTTTTGGCCAATACTGC | 60 |
| *OR18* | TTGGATGCTGTTTCGCAAGC | TCCATGCGCCTCAACAAATC | 60 |
| *OR19* | AGAGCAGAGCAATCGAATGG | AGACGATGCGTTTGACGATC | 60 |
| *OR2* | TCAAAGGCCGCATCAAGTTC | AGCAGCAGTAACCGCAAAAC | 60 |
| *OR20* | GAAACCATTCGCAAGTTCGG | TGAACACGAACCAGCTCATG | 60 |
| *OR21* | TGGTTCGCCAATGAAGTCAC | AGCGCCACTTCCTTTTGAAC | 60 |
| *OR22* | TTCGAAGACTGGCAGCATTG | TACGGCGTCACAATAAGGTG | 60 |
| *OR23* | TAGCGCTAATGGCACCTTTC | TCATCACGCAATCACCGTAG | 60 |
| *OR24* | TATGGCAGCCATTTGCGTTG | TCCCACTTGCATTGCATCAC | 60 |
| *OR25* | TTGTGGACCGCATTAAGCAG | CACTTCTCCATGGTTGAATGCC | 60 |
| *OR26* | AGCGAGTCGGTGAATCAATC | TCACAGCACTGCATCATCAC | 60 |
| *OR27* | CGTTAGTGATTTGCCTGACTGG | TTGCAGTTGTACACCGCATC | 60 |
| *OR28* | ATTCAAAGCCAAGGCATCGC | TATAGAAAGCAGCGCCATGC | 60 |
| *OR29* | AAAATCCTAGCGGCTCAAGC | ACGCGCTGATGTATATGCTG | 60 |
| *OR3* | TTGGAATGTGACGCCAACAC | ATAGCATGGCTCGCTTCTTC | 60 |
| *OR30* | GCGAGAAATTTCGTCAAGCG | CCCTCATCAGCATGAACAACAC | 60 |
| *OR31* | TAGGAACTGGGTCCGAAGTTTC | GTACAGGAACACTTGCAATGCC | 60 |
| *OR32* | AGCTGCAAAACGTGTATGGC | AGGGAAGGAAAATGTGGAGGAC | 60 |
| *OR33* | TAACGTTTTCACCGGGATGG | AAACGGTCAACATGGACGAG | 60 |
| *OR34* | ATGATGCGCCACTTGCTATC | ACAAATGGCCAAGCTCGAAG | 60 |
| *OR35* | ACCAGTCACAAAGTCCGTCAG | ACGCCGTTGAAGATGATCAC | 60 |
| *OR36* | TTGCGTTGGTAATGCTGGTG | GATGATGGTCATGAAGCTAGCG | 60 |
| *OR37* | TATTCTACTGTGGCTGGCAGTC | TGCATGATCGCCAACAGTAC | 60 |
| *OR38* | AAGCGAAATAGGCGACGATC | TCGTGGCAGCCATTATTTCG | 60 |
| *OR39* | TTCATAGCCAAGACCCAGAGAC | TGACCTTGGTGAATGTCTCCAG | 60 |
| *OR4* | AAGCCACACGATGAACATGC | ACAAATGAGTGTGCCGCTAG | 60 |
| *OR40* | TGGTGTGGGCATGAGCTTAC | ATGGCAAAGCATAGCTCTCG | 60 |
| *OR41* | ACCTGCAGAGCTACTTCATGAG | TGCGTTTGCTGAGTACATCG | 60 |
| *OR42* | TAGTCGAAAGAGCGAACAGACC | CGCCGTAAAAGTTGCAAGTG | 60 |
| *OR43* | TGTTTTGTCTGCTGGGCATC | TCTTATCATGGTGCCGAGATGG | 60 |
| *OR44* | AGTTTGTGACGCATGTTGGG | AGGGAGCGTTGCATAATGAG | 60 |
| *OR45* | CGCGACGACATCATCATCATC | AGTCTCAAGCTGCACATGTG | 60 |
| *OR46* | ACGCAACATCAACCTTGTGG | GCGCACAACCGAAACAAAAG | 60 |
| *OR47* | GCTGGTGCTGGTCATGGAG | ATCCATTGGATTGGTGTCGAG | 60 |
| *OR5* | TCTGGCTGCATTTCAGTGTG | ACTGAAAATGCGTCCTCCAG | 60 |
| *OR6* | ACGTCGTTGCTGCGAAAATG | AGAGATGTTGTGCGTCATGC | 60 |
| *OR7* | ACCACGTGTTTGCACTCTTC | ACAGCTTGTCCATGTTGCTC | 60 |
| *OR8* | ACAACATGGTGCGTCTGTTG | TCTTCCGACAACTGATTGCC | 60 |
| *OR9* | TTTACCGCGCTTTCACACTC | TGGAACACCACTTGCTTTGC | 60 |
| *ORCO* | AAAATGCCGGCATGTCGAAC | TCAGCGCAAAGAATCCAAGC | 60 |
| *PBP1* | AATGCGTCTCGTGTGTTTGG | TGAGCTCATCTTCGTGAGTAGC | 60 |
| *PBP2* | AAGCCTTTGGAAGACTGCAG | TGTTTCTGCGCAAACTCCTG | 60 |
| *PBP3* | AGAAGTTCGAGCGAGATGACG | ACATGTCTGTGAGGACTTCGG | 60 |
| *PBP4* | ACGAGTTCTTGAGCCCTGATG | AGAAGATCCACCAAGCGGTTAG | 60 |
| *PBP5* | ACTGTGCAAGAGACAGACGAG | TTCATACGTCTCCTGTGACCTG | 60 |
| *PR1* | TGATCCCAATGTACGCCAAC | AAAAACGTGCCGTACCAACC | 60 |
| *PR2* | TTGCTTTTTCGAGGGCACTG | TTGTTGTACATCGGCGTCAG | 60 |
| *PR3* | AGCAGGTATCTGAGAGATTGGC | AGCTTGCGTTGTCATCTGTG | 60 |
| *PR4* | AGATGCGGTATACGGTTTGC | AACATTGACAACGCCCAAGG | 60 |
| *AK* | TCTTGGTGTGGTGCAATGAG | AAGGAACGCGCTTCTCAATG | 60 |
| *Actin* | ATCAAGGAGAAGCTCTGCTACG | ATCGTTCGTTTCCGATGGTG | 60 |
