# Supplementary material for: Age influences the olfactory profiles of the migratory oriental armyworm mythimna separate at the molecular level
Source: BMC Genomics. 2017 Jan 5;18:32. doi: 10.1186/s12864-016-3427-2 (PMC5217624; doi:10.1186/s12864-016-3427-2)

Fig. S1, Size distribution of all 41,056 unigenes assembled from the pooled *M. separata* RNA extract

Fig.S2, Aligned putative full ORF of OBP gene sequences of *M. separata*. Six conserved cysteines were highlighted by blue color.


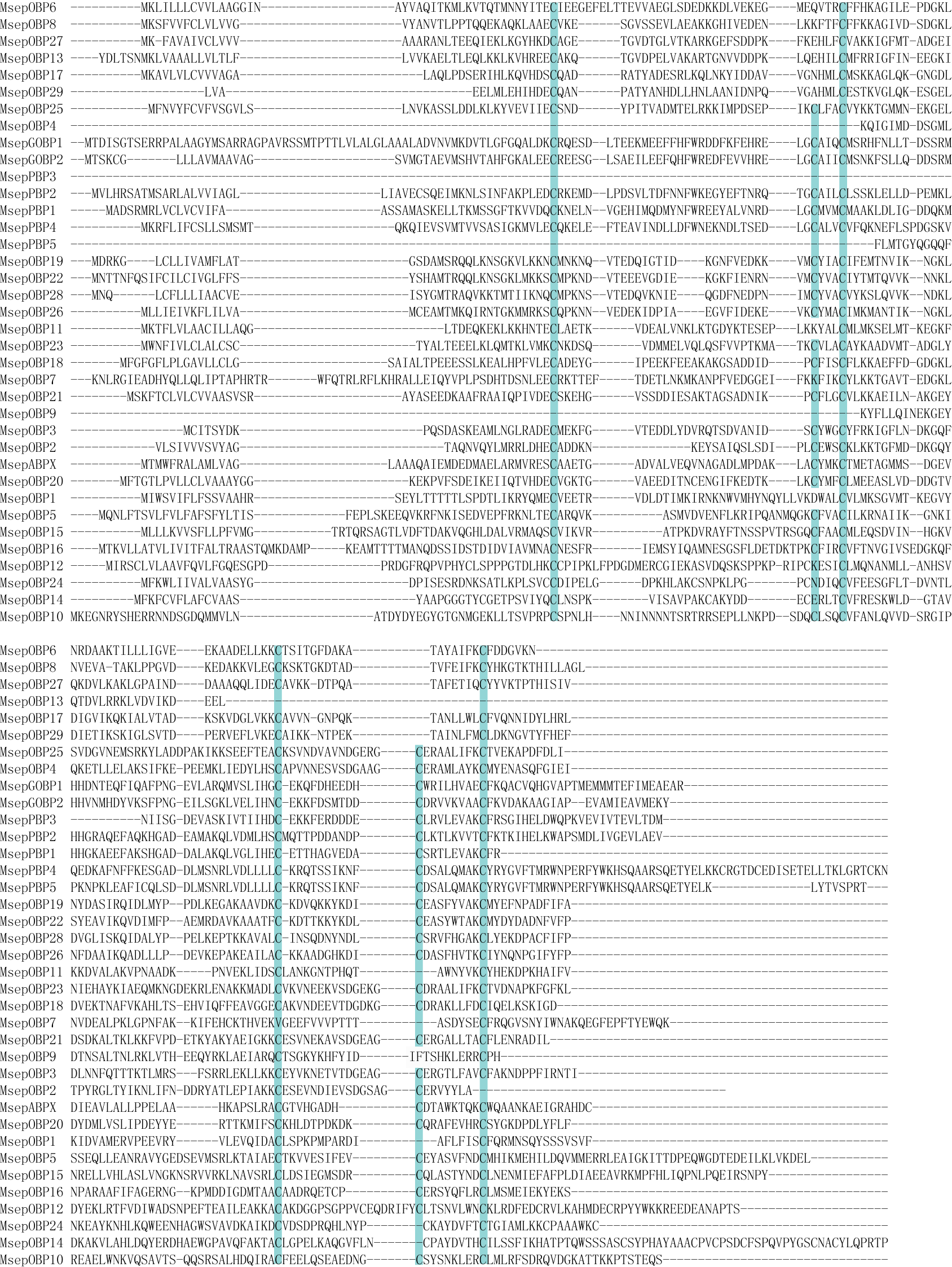


Fig.S3, Aligned putative ORF of CSP gene sequences of *M. separata*, CSP13 and CSP14 excluded. Four conserved cysteines were highlighted by blue color


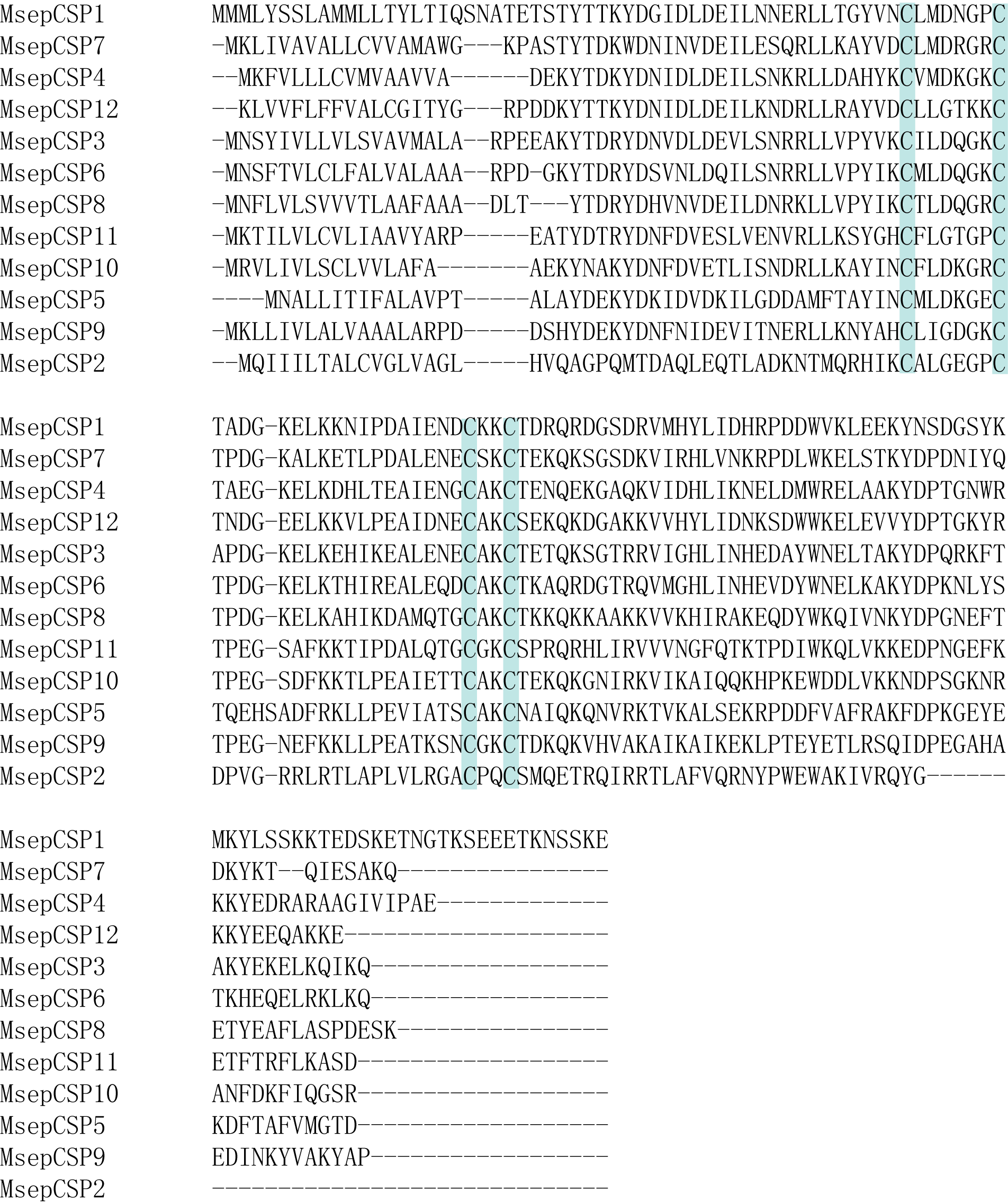

Supplement: Additional file 2: Figure S1. — Size distribution of all 41,056 unigenes assembled from the pooled M. separata RNA extract. Figure S2. Aligned putative full ORF of OBP gene sequences of M. separata. Six conserved cysteines were highlighted by blue color. Figure S3. Aligned putative ORF of CSP gene sequences of M. separata, CSP13 and CSP14 excluded. Four conserved cysteines were highlighted by blue color. (DOC 683 kb) [file 12864_2016_3427_MOESM2_ESM.doc]
